# Matching-Adjusted Indirect Comparisons of Filgotinib vs Vedolizumab, Tofacitinib, and Ustekinumab for Moderately to Severely Active Ulcerative Colitis

**DOI:** 10.1093/ibd/izad037

**Published:** 2023-03-22

**Authors:** Xiaoyan Lu, Zheng-Yi Zhou, Yiqiao Xin, Min-Jung Wang, Emma Gray, Vipul Jairath, James Oliver Lindsay

**Affiliations:** Galapagos NV, Romainville, France; Analysis Group, Boston, MA, USA; Analysis Group, Boston, MA, USA; Analysis Group, Boston, MA, USA; Analysis Group, Boston, MA, USA; Schulich School of Medicine and Dentistry, Western University, London, ON, Canada; The Royal London Hospital, Barts Health NHS Trust, London, United Kingdom; Centre for Immunobiology, Blizard Institute, Queen Mary University of London, London, United Kingdom

**Keywords:** filgotinib, MAIC, ulcerative colitis

## Abstract

**Background:**

Where head-to-head trials are lacking, indirect comparative effectiveness can aid treatment decisions. We conducted matching-adjusted indirect comparisons of clinical outcomes with filgotinib vs recently approved comparators (vedolizumab, tofacitinib, ustekinumab) in patients with moderately to severely active ulcerative colitis (UC).

**Methods:**

Individual patient data from the SELECTION trial (NCT02914522) for filgotinib 200 mg were weighted to match average baseline characteristics of active treatment and placebo arms in comparator trials. Efficacy outcomes were compared for biologic-naive and biologic-experienced subgroups in induction and maintenance populations, if data were available. Safety and health-related quality of life outcomes were compared in the overall maintenance population.

**Results:**

Filgotinib had a similar effect on efficacy outcomes compared with tofacitinib, ustekinumab, and subcutaneous vedolizumab in both the induction and maintenance populations. Filgotinib showed improved clinical response vs intravenous (IV) vedolizumab (odds ratio, 2.4; 95% confidence interval [CI], 1.0 to 5.5; *P* < .05) among the biologic-experienced induction population, and improved corticosteroid-free clinical remission (odds ratio, 15.2; 95% CI, 1.6 to 139.9; *P* < .05) among the biologic-naive maintenance population. Improved efficacy outcomes were reported with filgotinib compared with ustekinumab among the maintenance population. Higher estimates of serious adverse events were reported for filgotinib compared with vedolizumab IV 300 mg and tofacitinib 5 mg; however, imbalances were noted in their placebo groups. Health-related quality of life outcomes were similar between filgotinib and comparators.

**Conclusions:**

Matching-adjusted indirect comparison results suggest superiority of filgotinib 200 mg over vedolizumab IV in terms of clinical response and corticosteroid-free clinical remission in certain patient populations, noting small sample sizes and wide CIs, which may aid the selection of advanced therapies for moderately to severely active UC. A potential increased risk of serious adverse events was reported for filgotinib 200 mg vs vedolizumab IV and tofacitinib 5 mg, but findings should be interpreted with caution owing to underlying imbalances observed between the placebo groups of SELECTION and comparator trials.

**Key Messages**

**What is already known?**

- In the absence of head-to-head trials, indirect analyses may aid treatment decisions through comparison of phase 3 trials of advanced therapies that demonstrated superiority over placebo.

**What is new here?**

- Matching-adjusted indirect comparisons of filgotinib with 3 approved ulcerative colitis treatments showed similar efficacy, safety, and health-related quality of life outcomes in patients with moderately to severely active ulcerative colitis, with some benefits in clinical response and corticosteroid-free clinical remission.

**How can this study help patient care?**

- This study provides insights into the relative efficacy, safety, and health-related quality of life outcomes of filgotinib compared with vedolizumab, tofacitinib, and ustekinumab, which may inform treatment and reimbursement decisions.

## Introduction

Ulcerative colitis (UC) is a chronic inflammatory disorder of the colonic mucosal surface most frequently presenting as bloody diarrhea. Other symptoms include urgency, incontinence, fatigue, abdominal pain, fever, and weight loss.^1^ The incidence and prevalence of the disease have been increasing over time, with an incidence of 19 to 25 cases per 100 000 person-years and a prevalence of 214 to 505 cases per 100 000 people in North America and northern Europe.^2^ At present, there is no cure for UC, and lifelong therapy is required, the aim of which is to induce and maintain clinical and endoscopic remission, prevent disease-related complications, and restore quality of life.^2,3^

The introduction of biologics in the past 2 decades has expanded the treatment landscape for UC.^1^ To date, biologic treatments for UC include anti-tumor necrosis factor α (anti-TNF) agents (infliximab [Remicade; Centocor BV], adalimumab [Humira; AbbVie Deutschland GmbH & Co.KG], and golimumab [Simponi; Janssen Biologics BV]), anti-integrin antibodies (vedolizumab [Entyvio; Takeda]), anti-interleukin-12/23 antibodies (ustekinumab [Stelara; Janssen-Cilag International NV]), and Janus kinase (JAK) inhibitors (tofacitinib [Xeljanz; Pfizer]). Both anti-TNF agents (infliximab, adalimumab, and golimumab) and non–TNF-targeting agents (vedolizumab, ustekinumab, and tofacitinib) are recommended as induction and maintenance therapies for patients with moderately to severely active UC according to several clinical guidelines.^4,5^ Although anti-TNF agents are commonly used as the first-line treatment, approximately half of patients with UC fail to respond to anti-TNF therapies or lose response over time, while some patients cannot tolerate anti-TNF agents.^6^ For these patients, non–TNF-targeting agents such as vedolizumab, ustekinumab, and tofacitinib are recommended.^4,5,7^

Filgotinib (Jyseleca; Galapagos) is a once-daily, orally administered, JAK1-preferential inhibitor developed for the treatment of inflammatory conditions, including rheumatoid arthritis, Crohn’s disease, and UC.^8^ Filgotinib is approved in the European Union (2021)^9^ and Japan (2022)^10^ for the treatment of moderately to severely active UC in adult patients. The efficacy and safety of filgotinib were demonstrated in the phase 2b/3, randomized, double-blind, placebo-controlled SELECTION trial (NCT02914522) in biologic-naive and biologic-experienced adult patients with moderately to severely active UC.^11^ Filgotinib 200 mg achieved all primary endpoints, inducing clinical remission, at the end of the induction period (week 10) and maintained clinical remission at the end of the maintenance period (week 58) in a significantly higher proportion of patients compared with placebo.^11^

Analyses of the comparative effectiveness of filgotinib relative to other interventions are needed to inform treatment and reimbursement decisions and cost-effectiveness analyses. In the absence of head-to-head clinical trials, indirect treatment comparisons can be used to compare clinical outcomes between treatments. While traditional network meta-analyses (NMAs) can be conducted to rank treatment efficacy, heterogeneity in patient populations across trials cannot be fully adjusted for using these analyses.

Alternatively, matching-adjusted indirect comparison (MAIC), which is a population-adjusted method of indirect treatment comparison, adjusts for differences in trial designs.^12,13^ Whereas traditional propensity score matching requires individual patient-level data (IPD) from all trials in the comparison, MAIC is a form of propensity score matching that uses IPD from one trial and published aggregate data from another trial.^12^ The MAIC approach adjusts for available baseline factors that are both imbalanced between the trials and suspected of modifying the treatment effect of the intervention relative to placebo.^12-14^

Our study compared efficacy, safety, and health-related quality of life (HRQoL) outcomes in patients with moderately to severely active UC using an anchor-based MAIC to compare filgotinib with each of its key comparators, intravenous (IV) vedolizumab, subcutaneous (SC) vedolizumab, tofacitinib, and ustekinumab, using data from the GEMINI 1, VISIBLE 1, OCTAVE, and UNIFI trials, respectively.^15-20^ Vedolizumab, tofacitinib, and ustekinumab are approved in the United States and Europe for the treatment of moderately to severely active UC as first-line treatment or subsequent therapy after inadequate response with, after loss of response to, or after intolerance to either biologic agents or conventional therapy.^21-26^ Notably, the sphingosine-1-phosphate subtype 1 receptor modulator ozanimod and the JAK inhibitor upadacitinib were not included in the comparisons made here because, at the time of the analysis, induction and maintenance phase data from these small-molecule drugs were not available.^27,28^ Furthermore, neither compound had received approval by the European Medicines Agency for use as therapy in UC at that time. Additionally, anti-TNF agents were not included in this analysis owing to differences in patient populations and study design, including the use of treat-through approaches; however, these have recently been evaluated using the NMA approach.^29-31^ The findings in this study provide additional evidence that may help to inform clinical decisions and treatment guidelines on the basis of the recently approved drugs.

## Methods

### Overview

IPD from the SELECTION trial were weighted using propensity scoring to match average baseline characteristics of the active treatment and placebo arms in comparator trials.^12^ Efficacy outcomes were compared separately for biologic-naive and biologic-experienced subgroups in both the induction and maintenance populations, if data were available. Safety and HRQoL outcomes were compared in the overall maintenance population owing to availability of data. The anchor-based MAIC was conducted following the general methodology described by Signorovitch et al^12,13^ and Phillippo et al.^14^

### Study Population and Data Source

IPD were obtained from the 58-week, phase 2b/3, randomized, double-blind, placebo-controlled SELECTION trial (NCT02914522) evaluating filgotinib 200 mg vs placebo among patients with moderately to severely active UC. Comparator trials included in the MAIC were those evaluating treatment with European Medicines Agency approval for UC and with data available at the time of this study; anti-TNF agents were not included owing to differences in population and study design. For all comparator drugs, aggregate data were extracted from trial publications or published documents from the National Institute for Health and Care Excellence, European Medicines Agency, or U.S. Food and Drug Administration. Only the treatment arms with a dose schedule and administration route recommended by the European Medicines Agency and with routine usage confirmed by a clinical expert were considered. These included vedolizumab 300 mg IV evaluated for induction and vedolizumab 300 mg IV every 8 weeks evaluated for maintenance in the 52-week, phase 3, randomized, placebo-controlled GEMINI 1 trial^16^; vedolizumab 108 mg SC every 2 weeks evaluated for maintenance in the 52-week, phase 3, randomized, placebo-controlled VISIBLE 1 trial (vedolizumab IV was given as induction treatment followed by vedolizumab SC as maintenance treatment)^18^; tofacitinib 10 mg twice daily evaluated for induction in the 8-week, randomized, placebo-controlled OCTAVE 1 and OCTAVE 2 trials; tofacitinib 5 mg twice daily evaluated for maintenance in the 52-week, randomized, placebo-controlled OCTAVE SUSTAIN trial^15,17,19^; and ustekinumab 6 mg/kg IV evaluated for induction and ustekinumab 90 mg SC every 8 weeks evaluated for maintenance in the 52-week, randomized, placebo-controlled UNIFI trial.^20^

### Baseline Characteristics Matched in the MAIC

A feasibility assessment was conducted to determine cross-trial similarities and differences. The study designs and study populations were generally similar between the SELECTION and comparator trials, therefore, matching on inclusion or exclusion criteria was not necessary for this analysis (**Supplementary Tables 1 and 2**). SELECTION trial IPD were reweighted to match the aggregated mean or proportion of baseline characteristics in each of the comparator trials for the active treatment and placebo arms, separately. Matched baseline characteristics were sex, age, weight, smoking status, total Mayo score, disease duration, concomitant corticosteroid use, and history of anti-TNF therapy failure (for the biologic-experienced induction population and maintenance population only), and were selected based on availability in both the SELECTION and comparator trials, imbalance of characteristics between the trials, and consultation with clinical experts. Owing to differences in reporting of baseline characteristics across the trials, the set of baseline characteristics that were matched on were specific to each comparison and are listed in Tables 1 to 4 and Supplementary Tables 3 to 7.

**Table 1.** Baseline characteristics of the overall maintenance population in the SELECTION (filgotinib) and GEMINI 1 (vedolizumab IV) trials, before and after matching.

Baseline matching variables	Before matching						After matching			
	SELECTION		GEMINI 1		*P* value A vs C^a^	*P* value B vs D^a^	SELECTION		GEMINI 1	
	A. Filgotinib (n = 202)	B. Placebo (n = 99)	C. Vedolizumab IV (n = 122)	D. Placebo (n = 126)			Filgotinib (n = 202, ESS = 106)	Placebo (n = 99, ESS = 55)	Vedolizumab IV (n = 122)	Placebo (n = 126)
**Male**	95 (47.0)	48 (48.5)	70 (57.4)	69 (54.8)	.09	.42	57.4	54.8	57.4	54.8
**Age, y**	43.1 ± 13.8	42.5 ± 13.0	41.0 ± 13.0	40.3 ± 14.0	.17	.23	41.0 ± 14.0	40.3 ± 11.6	41.0 ± 13.0	40.3 ± 14.0
**Current smoker**	13 (6.4)	1 (1.0)	7 (5.7)	8 (6.3)	.99	.08	5.7	6.3	5.7	6.3
**Weight, kg**	70.2 ± 18.4	72.4 ± 18.3	78.2 ± 19.0	74.7 ± 20.0	<.001^b^	.37	78.2 ± 20.8	74.7 ± 17.9	78.2 ± 19.0	74.7 ± 20.0
**Disease duration, y**	8.4 ± 7.4	8.9 ± 7.6	6.2 ± 5.0	7.8 ± 7.0	<.01^c^	.27	6.2 ± 5.8	7.8 ± 7.0	6.2 ± 5.0	7.8 ± 7.0
**Total Mayo score at induction baseline**	8.9 ± 1.3	8.9 ± 1.4	8.4 ± 1.8	8.4 ± 1.8	<.01^c^	<.05^d^	8.4 ± 1.3	8.4 ± 1.5	8.4 ± 1.8	8.4 ± 1.8
**Concomitant corticosteroid use at induction baseline**	76 (37.6)	39 (39.4)	70 (57.4)	72 (57.1)	<.01^c^	<.05^d^	57.4	57.1	57.4	57.1
**History of anti-TNF failure at induction baseline**	76 (37.6)	39 (39.4)	43 (35.2)	38 (30.2)	.76	.19	35.2	30.2	35.2	30.2

Values are n (%), %, or mean ± SD, unless otherwise indicated.

Abbreviations: ESS, effective sample size; IV, intravenous; TNF, tumor necrosis factor.

^a^ *P* values for continuous variables were calculated using the Wald test. *P* values for categorical variables were calculated using the chi-square test. The Fisher exact test was used for categorical variables with small frequency (ie, n < 5).

^b^ *P* value <.001.

^c^ *P* value <.01.

^d^ *P* value <.05.

In the induction population, matching was conducted in the biologic-naive and biologic-experienced induction populations in the GEMINI 1 and OCTAVE 1 and 2 trials. By contrast, in the UNIFI trial, matching used the overall induction population because baseline characteristics stratified by history of biologic treatment were not available. In the maintenance population, matching was conducted in the overall maintenance population for all comparisons owing to a lack of stratified baseline data.

### Outcome Measures

Outcomes compared for each trial depended on data availability and clinical interest in both IPD and aggregate data. Definitions of outcomes were compared between the SELECTION trial and each of the comparator trials. If definitions did not match, the IPD of the SELECTION trial were used to generate the outcome as defined in the comparator trial, if achievable (**Supplementary Table 8**).

Efficacy outcomes were compared at the end of the induction and maintenance phases between filgotinib and each comparator, and included clinical remission, endoscopic improvement, and clinical response for the induction phase as well as clinical remission, endoscopic improvement, clinical response, sustained clinical remission, and corticosteroid-free clinical remission for the maintenance phase. Comparisons were conducted separately among biologic-naive and biologic-experienced subgroups if data for the stratified populations were available. There were some differences in the classification of subgroups between the trials (biologic experienced vs anti-TNF experienced; biologic failure vs anti-TNF failure; biologic naive vs anti-TNF naive). Biologic-experienced subgroups were deemed to be comparable to anti-TNF–experienced/failure subgroups in the comparator trials, given the large overlap between anti-TNF and biologic use. Similarly, biologic-naive subgroups were deemed to be comparable to anti-TNF–naive subgroups in the comparator trials.

Safety (serious adverse events [AEs], AEs leading to treatment discontinuation, any infection, serious infections, and herpes zoster) and HRQoL outcomes (Inflammatory Bowel Disease Questionnaire [IBDQ] total score and 36-item Short-Form Health Survey [SF-36] score [Physical and Mental Component Summary scores]) were compared for the overall maintenance population only owing to data availability.

### Statistical Methods of MAIC

The MAIC approach used a propensity score model to assign weights to SELECTION trial patients to balance average baseline characteristics between trials.^12-14^ Effective sample size (ESS) after reweighting was calculated for SELECTION trial patients by assigned arm, which indicates the extent of overlap between SELECTION trial patients and patients from each comparator trial.

For binary outcomes, reweighted estimates of odds ratios (ORs) were made for comparing filgotinib vs placebo and comparator drug vs placebo. Risk differences (RDs) were estimated for safety outcomes when the ORs could not be computed (with 0% in one arm). For continuous outcomes, the reweighted difference in mean change between filgotinib vs placebo was compared against the corresponding difference in mean change between the comparator drug vs placebo to obtain the anchor-based estimate of the indirect treatment effect.

Comparisons of differences between filgotinib vs each comparator drug were conducted using Wald tests to determine if explanatory variables were significant, incorporating the weights obtained in the matching process. *P* values and the 95% confidence intervals (CIs) of the OR and RD were reported. Statistical significance was considered at a level of .05.

All analyses were conducted using R version 4.0.2 (R Foundation for Statistical Computing).

## Results

### Induction Phase

The IPD in the SELECTION trial included 382 biologic-naive patients (filgotinib 200 mg, n = 245; placebo, n = 137) and 404 biologic-experienced patients (filgotinib 200 mg, n = 262; placebo, n = 142) in the induction population. Baseline characteristics, both before and after matching, for the induction population are summarized in **Supplementary Tables 3 to 7**.

In both the biologic-naive and biologic-experienced subgroups, there were no significant differences in clinical response, endoscopic improvement, or clinical remission between filgotinib 200 mg and each comparator at the end of the induction phase, with the exception of a significant improvement in clinical response rate with filgotinib 200 mg compared with vedolizumab IV 300 mg in the biologic-experienced (anti-TNF failure) subgroup (OR, 2.4; 95% CI, 1.0 to 5.5; *P* < .05). Full results are presented in **Appendix 1** and **Supplementary Tables 9 to 11**.

### Maintenance Phase

#### Study population

IPD from the SELECTION trial from 301 patients (filgotinib 200 mg, n = 202; placebo, n = 99) in the maintenance population were matched with aggregate data from the maintenance populations of GEMINI 1 (248 patients: vedolizumab IV 300 mg, n = 122; placebo, n = 126), VISIBLE 1 (162 patients: vedolizumab SC 108 mg, n = 106; placebo, n = 56), OCTAVE SUSTAIN (396 patients: tofacitinib 5 mg, n = 198; placebo, n = 198), and UNIFI (351 patients: ustekinumab 90 mg, n = 176; placebo, n = 175) trials.

#### Baseline characteristics

After matching, baseline characteristics were balanced between the maintenance populations of the SELECTION and GEMINI 1 (vedolizumab IV 300 mg [Table 1]; ESS for SELECTION, 161 patients: filgotinib 200 mg, n = 106; placebo, n = 55), VISIBLE 1 (vedolizumab SC 108 mg [Table 2]; ESS for SELECTION, n = 234 patients: filgotinib 200 mg, n = 149; placebo, n = 85), OCTAVE SUSTAIN (tofacitinib 5 mg [Table 3]; ESS for SELECTION, n = 257 patients: filgotinib 200 mg, n = 178; placebo, n = 79), and UNIFI (ustekinumab 90 mg [Table 4]; ESS for SELECTION, n = 238 patients: filgotinib 200 mg, n = 157; placebo, n = 81) trials.

**Table 2.** Baseline characteristics of the overall maintenance population in the SELECTION (filgotinib) and VISIBLE 1 (vedolizumab SC) trials, before and after matching.

Baseline matching variables	Before matching						After matching			
	SELECTION		VISIBLE 1				SELECTION		VISIBLE 1	
	A. Filgotinib (n = 202)	B. Placebo (n = 99)	C. Vedolizumab SC (n = 106)	D. Placebo (n = 56)	*P* value A vs C^a^	*P* value B vs D^a^	Filgotinib (n = 202, ESS = 149)	Placebo (n = 99, ESS = 85)	Vedolizumab SC (n = 106)	Placebo (n = 56)
**Male**	95 (47.0)	48 (48.5)	65 (61.3)	34 (60.7)	**<.05** ^b^	.19	61.3	60.7	61.3	60.7
**Age, y**	43.1 ± 13.8	42.5 ± 13.0	38.1 ± 13.1	39.4 ± 11.7	**<.01** ^c^	.13	38.1 ± 12.8	39.4 ± 12.9	38.1 ± 13.1	39.4 ± 11.7
**Current smoker**	13 (6.4)	1 (1.0)	11 (10.4)	0 (0.0)	.32	1.00	10.4	0.0	10.4	0.0
**Weight, kg**	70.2 ± 18.4	72.4 ± 18.3	71.6 ± 17.2	74.0 ± 20.9	.51	.64	71.6 ± 17.8	74.0 ± 18.2	71.6 ± 17.2	74.0 ± 20.9
**Disease duration, y**	8.4 ± 7.4	8.9 ± 7.6	8.0 ± 6.2	7.4 ± 7.1	.62	.22	8.0 ± 7.1	7.4 ± 6.8	8.0 ± 6.2	7.4 ± 7.1
**Total Mayo score: 6-8**	74 (36.6)	35 (35.4)	46 (43.4)	20 (35.7)	.30	1.00	43.4	35.7	43.4	35.7
**Total Mayo score: 9-12**	128 (63.4)	64 (64.6)	60 (56.6)	36 (64.3)	.30	1.00	56.6	64.3	56.6	64.3
**Concomitant corticosteroid use at induction baseline**	78 (38.6)	40 (40.4)	45 (42.5)	24 (42.9)	.60	.90	42.5	42.9	42.5	42.9
**History of anti-TNF failure at induction baseline**	76 (37.6)	39 (39.4)	39 (36.8)	19 (33.9)	.98	.62	36.8	33.9	36.8	33.9

Values are n (%), %, or mean ± SD, unless otherwise indicated.

Abbreviations: ESS, effective sample size; SC, subcutaneous; TNF, tumor necrosis factor.

^a^ *P* values for continuous variables were calculated using the Wald test. *P* values for categorical variables were calculated using the chi-square test. The Fisher exact test was used for categorical variables with small frequency (ie, n < 5).

^b^ *P* value <.05.

^c^ *P* value <.01.

**Table 3.** Baseline characteristics of the overall maintenance population in the SELECTION (filgotinib) and OCTAVE SUSTAIN (tofacitinib) trials, before and after matching.

Baseline matching variables	Before matching						After matching			
	SELECTION		OCTAVE SUSTAIN				SELECTION		OCTAVE SUSTAIN	
	Filgotinib (n = 202)	B. Placebo (n = 99)	C. Tofacitinib (n = 198)	D. Placebo (n = 198)	*P* value A vs C^a^	*P* value B vs D^a^	Filgotinib (n = 202, ESS = 178)	Placebo (n = 99, ESS = 79)	Tofacitinib (n = 198)	Placebo (n = 198)
**Male**	95 (47.0)	48 (48.5)	103 (52.0)	116 (58.6)	.37	.13	52.0	58.6	52.0	58.6
**Age, y**	43.1 ± 13.8	42.5 ± 13.0	41.9 ± 13.7	43.4 ± 14.0	.38	.57	41.9 ± 13.8	43.4 ± 12.6	41.9 ± 13.7	43.4 ± 14.0
**Never smoked**	136 (67.3)	72 (72.7)	142 (71.7)	113 (57.1)	.40	**<.05** ^b^	71.7	57.1	71.7	57.1
**Weight at maintenance baseline, kg**	71.2 ± 18.3	73.0 ± 18.1	73.4 ± 17.8	76.2 ± 16.7	.23	.14	73.4 ± 18.5	76.2 ± 18.6	73.4 ± 17.8	76.2 ± 16.7
**Disease duration, y**	8.4 ± 7.4	8.9 ± 7.6	8.3 ± 7.2	8.8 ± 7.5	.90	.93	8.3 ± 7.4	8.8 ± 7.6	8.3 ± 7.2	8.8 ± 7.5
**Total Mayo score at maintenance baseline**	3.6 ± 1.9	3.3 ± 2.0	3.3 ± 1.8	3.3 ± 1.8	.14	.98	3.3 ± 2.0	3.3 ± 1.9	3.3 ± 1.8	3.3 ± 1.8
**Concomitant corticosteroid use at maintenance baseline**	80 (39.6)	40 (40.4)	103 (52.0)	105 (53.0)	**<.05** ^b^	.05	52.0	53.0	52.0	53.0
**History of anti-TNF failure at induction baseline**	76 (37.6)	39 (39.4)	83 (41.9)	89 (44.9)	.44	.43	41.9	44.9	41.9	44.9

Values are n (%), %, or mean ± SD, unless otherwise indicated.

Abbreviations: ESS, effective sample size; TNF, tumor necrosis factor.

^a^ *P* values for continuous variables were calculated using the Wald test. *P* values for categorical variables were calculated using the chi-square test. The Fisher exact test was used for categorical variables with small frequency (ie, n < 5).

^b^ *P* value <.05.

**Table 4.** Baseline characteristics of the overall maintenance population in the SELECTION (filgotinib) and UNIFI (ustekinumab) trials, before and after matching.

Baseline matching variables	Before matching						After matching			
	SELECTION		UNIFI				SELECTION		UNIFI	
	A. Filgotinib (n = 202)	B. Placebo (n = 99)	C. Ustekinumab (n = 176)	D. Placebo (n = 175)	*P* value A vs C^a^	*P* value B vs D^a^	Filgotinib (n = 202, ESS = 157)	Placebo (n = 99, ESS = 81)	Ustekinumab (n = 176)	Placebo (n = 175)
**Male**	95 (47.0)	48 (48.5)	94 (53.4)	107 (61.1)	.26	.06	53.4	61.1	53.4	61.1
**Age, y**	43.1 ± 13.8	42.5 ± 13.0	39.5 ± 13.3	42.0 ± 13.9	**<.01** ^b^	.78	39.5 ± 13.5	42.0 ± 12.8	39.5 ± 13.3	42.0 ± 13.9
**Weight, kg**	70.2 ± 18.4	72.4 ± 18.3	72.0 ± 19.1	71.7 ± 14.6	.35	.74	72.0 ± 18.5	71.7 ± 17.7	72.0 ± 19.1	71.7 ± 14.6
**Disease duration, y**	8.4 ± 7.4	8.9 ± 7.6	8.1 ± 6.6	7.5 ± 6.8	.69	.13	8.1 ± 7.1	7.5 ± 6.8	8.1 ± 6.6	7.5 ± 6.8
**Total Mayo score at induction baseline**	8.9 ± 1.3	8.9 ± 1.4	8.9 ± 1.6	8.7 ± 1.5	.92	.21	8.9 ± 1.3	8.7 ± 1.4	8.9 ± 1.6	8.7 ± 1.5
**Concomitant corticosteroid use at induction baseline**	78 (38.6)	40 (40.4)	95 (54.0)	95 (54.3)	**<.01** ^b^	**<.05** ^c^	54.0	54.3	54.0	54.3
**History of biologic failure at induction baseline**	85 (42.1)	41 (41.4)	91 (51.7)	88 (50.3)	.08	.20	51.7	50.3	51.7	50.3

Values are n (%), %, or mean ± SD, unless otherwise indicated.

Abbreviations: ESS, effective sample size.

^a^ *P* values for continuous variables were calculated using the Wald test. *P* values for categorical variables were calculated using the chi-square test. The Fisher exact test was used for categorical variables with small frequency (ie, n < 5).

^b^ *P* value <.01.

^c^ *P* value <.05.

#### Efficacy outcomes

##### Filgotinib vs Vedolizumab

**Figure 1.**
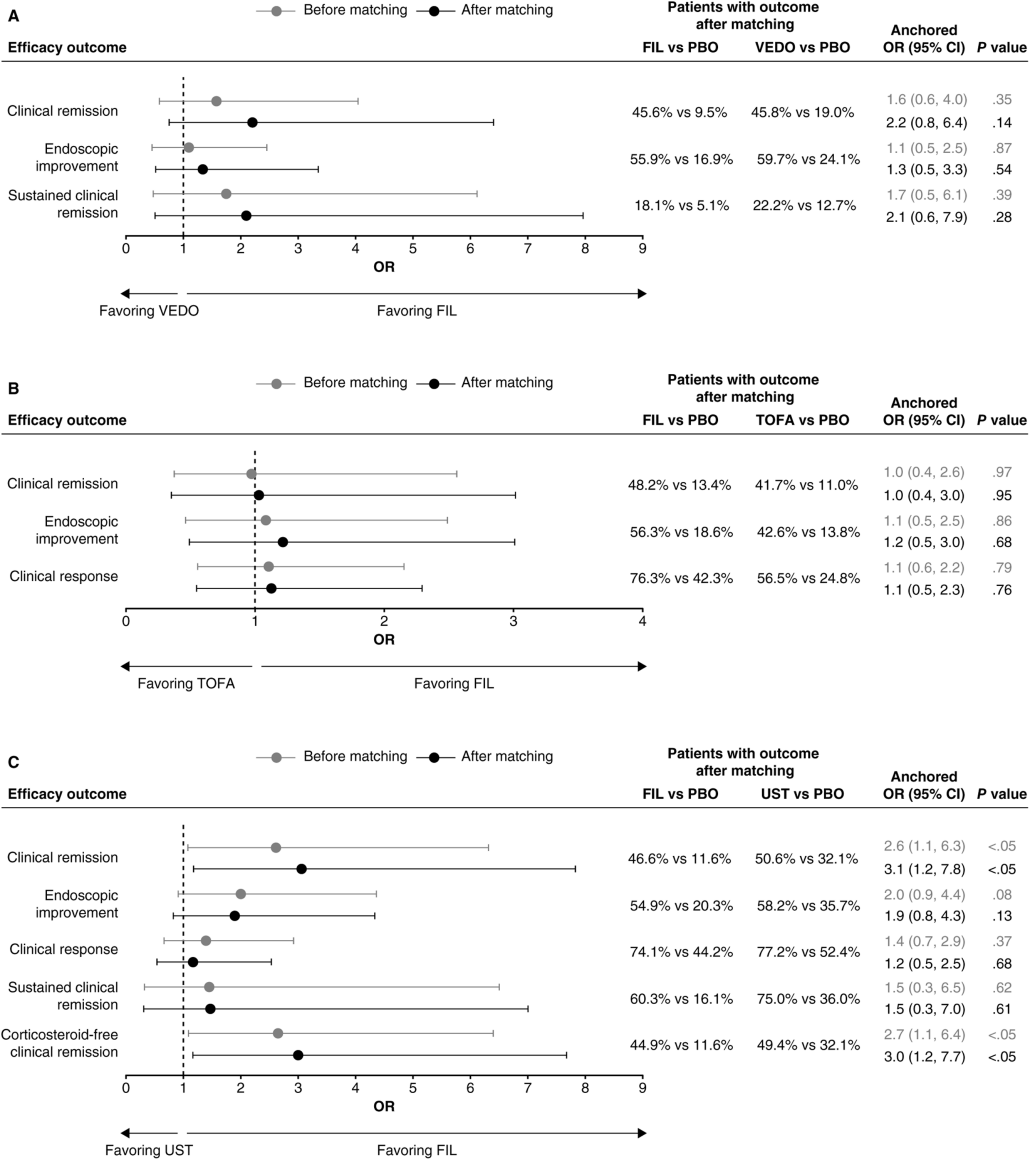
Efficacy outcomes among biologic-naive maintenance populations of filgotinib (FIL) vs (A) vedolizumab (VEDO) intravenous (IV), (B) tofacitinib (TOFA), and (C) ustekinumab (UST) at the end of the maintenance phase. The definitions of efficacy outcomes are reported in **Supplementary Table 8**. Corticosteroid-free clinical remission is not presented in the comparison of FIL with VEDO IV owing to wide confidence intervals [CIs] (odds ratio [OR], 15.2; 95% CI, 1.6 to 139.9). PBO, placebo.

After matching, clinical remission, endoscopic improvement, and sustained clinical remission were not significantly different between filgotinib 200 mg and vedolizumab IV 300 mg in either the biologic-naive or the biologic-experienced subgroups at the end of the maintenance phase (Figures 1A**and**2A). Filgotinib 200 mg was associated with significantly improved corticosteroid-free clinical remission compared with vedolizumab IV 300 mg (OR, 15.2; 95% CI, 1.6 to 139.9; *P* < .05) in the biologic-naive subgroup at the end of the maintenance phase.

**Figure 2.**
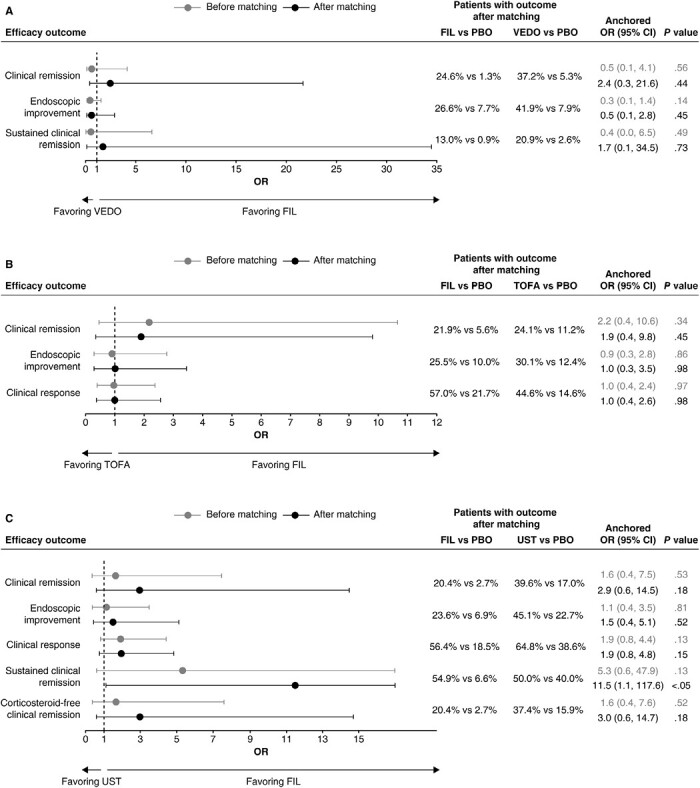
Efficacy outcomes among biologic-experienced subgroups of filgotinib (FIL) vs (A) vedolizumab (VEDO) intravenous (IV), (B) tofacitinib (TOFA), and (C) ustekinumab (UST) at the end of the maintenance phase. The definitions of efficacy outcomes are reported in **Supplementary Table 8**. Corticosteroid-free clinical remission is not presented in the comparison of FIL with VEDO IV owing to wide confidence intervals (CIs) (odds ratio [OR], 4.7; 95% CI, 0.2 to 93.2). PBO, placebo.

When compared with vedolizumab SC 108 mg, filgotinib 200 mg showed no significant difference in clinical remission, endoscopic improvement, sustained clinical remission, or corticosteroid-free clinical remission in either the biologic-naive or the biologic-experienced subgroup at the end of the maintenance phase (Table 5).

**Table 5:** ORs for efficacy outcomes among biologic-naive and biologic-experienced subgroups of filgotinib and vedolizumab SC at the end of the maintenance phase.

Filgotinib 200 mg vs vedolizumab SC	Biologic naive						Biologic experienced					
	Before matching			After matching			Before matching			After matching		
	OR	95% CI	*P* value^a^	OR	95% CI	*P* value^a^	OR	95% CI	*P* value^a^	OR	95% CI	*P* value^a^
**Clinical remission** ^b^	1.1	0.4 to 3.3	.81	1.3	0.4 to 3.9	.66	0.6	0.1 to 7.3	.73	1.4	0.1 to 17.1	.77
**Endoscopic improvement** ^c^	1.3	0.5 to 3.1	.62	1.2	0.5 to 3.1	.67	0.2	0.0 to 1.5	.12	0.2	0.0 to 1.9	.16
**Corticosteroid-free clinical remission** ^d^	2.8	0.2 to 46.2	.47	3.8	0.2 to 63.8	.36	1.2	0.1 to 21.8	.88	3.2	0.2 to 60.5	.44
	**OR**	**95% CI**	***P* value** ^a^	**OR**	**95% CI**	***P* value** ^a^	**RD**	**95% CI**	***P* value** ^a^	**RD**	**95% CI**	***P* value** ^a^
**Sustained clinical remission** ^e^	1.0	0.2 to 5.0	.96	1.2	0.2 to 5.8	.85	2.7	-5.8 to 11.3	.53	5.1	-4.4 to 14.6	.29

Abbreviations: CI, confidence interval; OR, odds ratio; RD, risk difference; SC, subcutaneous.

^a^ *P* values were calculated using the Wald test.

^b^Clinical remission is defined as the proportion of patients with total Mayo score ≤2 and no subscore >1 at the end of induction.

^c^Endoscopic improvement is defined as the proportion of patients with endoscopic subscore ≤1 at the end of induction.

^d^Corticosteroid-free clinical remission is defined as the proportion of patients using oral corticosteroids at induction baseline who discontinued corticosteroids and were in clinical remission at the end of maintenance.

^e^Sustained clinical remission is defined as the proportion of patients with clinical remission at the end of both induction and maintenance phases.

##### Filgotinib vs Tofacitinib

After matching, clinical remission, endoscopic improvement, and clinical response were not significantly different between filgotinib 200 mg and tofacitinib 5 mg in either the biologic-naive or the biologic-experienced subgroup at the end of the maintenance phase (Figures 1B**and**2B), and the odds of corticosteroid-free clinical remission were not significantly different between filgotinib 200 mg and tofacitinib 5 mg in the overall maintenance population (**Supplementary Table 12**). However, it should be noted that patients in the biologic-naive placebo subgroup of the SELECTION trial had higher odds of achieving clinical response than the same population of the OCTAVE SUSTAIN trial (OR, 2.2; 95% CI, 1.3 to 3.9; *P* < .01), which may suggest that differences in trial populations or design may not have been adjusted for in full.

##### Filgotinib vs Ustekinumab

In the biologic-naive subgroup, endoscopic improvement, clinical response, and sustained clinical remission were not significantly different between filgotinib 200 mg and ustekinumab 90 mg at the end of the maintenance phase; however, filgotinib 200 mg was associated with significantly better clinical remission (OR, 3.1; 95% CI, 1.2 to 7.8; *P* < .05) and corticosteroid-free clinical remission (OR, 3.0; 95% CI, 1.2 to 7.7; *P* < .05) compared with ustekinumab 90 mg (Figure 1C). Similarly, in the biologic-experienced (anti-TNF failure) subgroup, there was no significant difference in clinical remission, endoscopic improvement, clinical response, or corticosteroid-free clinical remission between filgotinib 200 mg and ustekinumab 90 mg at the end of the maintenance phase; however, filgotinib 200 mg was associated with significantly better sustained clinical remission (OR, 11.5; 95% CI, 1.1 to 117.6, *P* < .05) compared with ustekinumab 90 mg after matching (Figure 2C).

Despite the benefit shown with filgotinib 200 mg, it should be noted that the biologic-naive placebo subgroup of the SELECTION trial had a significantly lower proportion of patients who achieved clinical remission (OR, 0.3; 95% CI, 0.1 to 0.6; *P* < .01), endoscopic improvement (OR, 0.5; 95% CI, 0.2 to 0.9; *P* < .05), or corticosteroid-free clinical remission (OR, 0.3; 95% CI, 0.1 to 0.6; *P* < .01) compared with the same population of the UNIFI trial. Additionally, patients in the biologic-experienced (anti-TNF failure) placebo subgroup of SELECTION had significantly lower odds of all efficacy outcomes (clinical remission [OR, 0.1; 95% CI, 0.0 to 0.6; *P* < .01], endoscopic improvement [OR, 0.3; 95% CI, 0.1 to 0.8; *P* < .05], clinical response [OR, 0.4; 95% CI, 0.2 to 0.8; *P* < .05], sustained clinical remission [OR, 0.1; 95% CI, 0.0 to 0.8, *P* < .05], and corticosteroid-free clinical remission [OR, 0.1; 95% CI, 0.0 to 0.6; *P* < .05]) compared with the same population of the UNIFI trial.

#### Safety outcomes

We report safety outcomes in the overall maintenance populations. Filgotinib 200 mg and the comparators showed generally similar rates of AEs (**Supplementary Tables 13-16**). Overall, there was no significant difference observed between filgotinib 200 mg and the comparators in terms of the proportion of patients with AEs leading to treatment discontinuation, any infection, serious infections, or herpes zoster. However, filgotinib 200 mg was associated with significantly higher odds of serious AEs compared with vedolizumab IV 300 mg (OR, 11.8; 95% CI, 2.5 to 54.8; *P* < .01) and tofacitinib 5 mg (risk difference, 6.2; 95% CI, 0.6 to 11.8; *P* < .05). However, as reported with other outcomes there were significant differences in the likelihood of AEs between the placebo populations in the SELECTION trial and both the GEMINI 1 (OR, 0.1; 95% CI, 0.0 to 0.3; *P* < .001) and OCTAVE SUSTAIN (risk difference, -6.6; 95% CI, -10.0 to -3.1; *P* < .001) trials, meaning that the significant differences in the risk of serious AEs between filgotinib 200 mg vs vedolizumab IV 300 mg and tofacitinib 5 mg should be interpreted with caution.

#### HRQoL outcomes in the overall maintenance populations

In the overall maintenance population postmatching, filgotinib 200 mg was comparable with all interventions for all IBDQ and SF-36 outcomes. However, significant differences (*P* < .05) or differences close to significance (*P* = .05) were observed between the placebo population of the SELECTION trial and those of the GEMINI 1, OCTAVE SUSTAIN, and UNIFI trials for all assessed outcomes. It was not possible to verify if the placebo arms in the SELECTION and VISIBLE 1 trials were comparable after matching owing to the lack of arm-level data. Detailed results are presented in **Appendix 4** and **Supplementary Tables 17 to 20**.

## Discussion

This study is the first to present population-adjusted indirect comparisons of filgotinib, a once-daily, oral, JAK1-preferential inhibitor, with existing non–TNF-targeting treatments for patients with moderately to severely active UC. We used MAIC methodology to compare adjusted IPD on filgotinib derived from the pivotal phase 3 SELECTION trial with aggregate data on each of 4 contemporaneous interventions (vedolizumab IV, vedolizumab SC, tofacitinib, and ustekinumab). For vedolizumab, both IV and SC methods of administration were considered because some patients may prefer the convenience of self-administrating the more recently developed vedolizumab SC as long-term maintenance treatment for UC.^18^

Overall, filgotinib had a broadly similar efficacy, safety, and HRQoL profile to vedolizumab (IV and SC), tofacitinib, and ustekinumab, among both biologic-naive and biologic-experienced subgroups, during the induction and maintenance phases. Nonetheless, a few differences were observed in some comparisons of efficacy outcomes. Possible benefits of filgotinib 200 mg included significantly improved clinical response and corticosteroid-free clinical remission vs vedolizumab IV 300 mg, which may suggest a more rapid onset of clinical response with filgotinib 200 mg. However, these results should be interpreted with caution given the small sample sizes and wide CIs. Clinical remission, corticosteroid-free remission, and sustained clinical remission were also improved with filgotinib 200 mg compared with ustekinumab 90 mg. However, significant differences were noted between the placebo arm of the SELECTION trial and those of UNIFI in these efficacy outcomes, which may be due to differences in mode of action or dose regimen. Therefore, these results should also be interpreted with caution. Rates of AEs leading to treatment discontinuation, any infection, serious infections, and herpes zoster were similar between filgotinib 200 mg and its comparators. Significant increases in the proportion of patients experiencing serious AEs were reported for filgotinib compared with vedolizumab IV 300 mg or tofacitinib 5 mg. However, significant differences were identified between the placebo arm of the SELECTION trial and those of the GEMINI 1 and OCTAVE SUSTAIN trials in serious AEs (vs vedolizumab IV 300 mg [GEMINI 1] or tofacitinib 5 mg [OCTAVE SUSTAIN]). IBDQ and SF-36 outcomes were similar between filgotinib 200 mg and vedolizumab IV 300 mg or tofacitinib 5 mg, and mostly similar between filgotinib 200 mg and ustekinumab 90 mg. However, significant differences were identified between the placebo arm of the SELECTION trial and those of the GEMINI 1, OCTAVE SUSTAIN, and UNIFI trials in HRQoL outcomes (vs vedolizumab IV 300 mg [GEMINI 1], tofacitinib 5 mg [OCTAVE SUSTAIN], or ustekinumab 90 mg [UNIFI]). Unobserved factors in trial design and population may account for the differences observed between the placebo groups of the SELECTION and OCTAVE SUSTAIN trials. Notably, patients in the placebo group of the SELECTION trial had received filgotinib 200 mg during induction while the placebo group of the OCTAVE SUSTAIN trial may have received placebo during induction, owing to a difference in trial design.

Differences between the placebo groups included in anchored analyses have been identified previously, such as after matching in comparisons of treatments for psoriasis and epilepsy.^12,32^ In our study, the differences reported may be attributed to the heterogeneity in trial design, including variations in the duration of treatment and the protocol of defining AEs. The carryover effect of drugs taken by patients during the induction period may also have contributed to discrepancies seen in the placebo groups during the maintenance phase. Compounds with a different half-life and mode of action may potentially differ in their duration of biological effect upon cessation of therapy, which may consequently affect efficacy rates in placebo groups during the maintenance phase.^9,21-22^ For example, patients in the placebo groups in the UNIFI and SELECTION trials had received ustekinumab IV and filgotinib, respectively, at induction. The odds of achieving any of the efficacy outcomes at maintenance were consistently lower in the placebo group in the SELECTION trial than in the placebo group in the UNIFI trial, suggesting a potential carryover effect for ustekinumab.

Despite matching on key prognostic factors, effect modifiers may remain that are unquantifiable or unobserved, meaning that resultant heterogeneity may be attributed to the study design or the evolving treatment landscape, which can be accounted for only in head-to-head randomized trials. The residual effect of these factors may explain the differences observed in the placebo groups across trials, highlighting some of the limitations of indirect treatment comparisons, which need to be considered when interpreting the outcomes. Further validation through clinical trials or real-world evidence comparing these treatment options is needed.

Findings from this study are consistent with results from published NMAs. Lasa et al^31^ showed no significant differences in the efficacy (clinical remission and endoscopic improvement) of filgotinib 200 mg compared with induction or maintenance vedolizumab, tofacitinib, or ustekinumab in patients with moderately to severely active UC. Furthermore, no significant differences were observed among these active treatments in the maintenance of steroid-free remission and safety outcomes.^31^ Similarly, Lu et al^33,34^ reported no significant differences between filgotinib and other treatments, including vedolizumab, tofacitinib, and ustekinumab, regardless of biologic status. Nonetheless, numerically higher likelihoods were observed for filgotinib in achieving SF-36 Mental Component Summary response and remission, compared with other interventions.^33,34^

A key strength of our study was the ability to adjust for observed cross-trial differences using a broad list of patients’ baseline characteristics. The list of characteristics was selected based on discussions with clinical experts and the availability of these data from the clinical trials included in this analysis. By adjusting for cross-trial differences, the MAIC approach ensured a sufficient sample size used for the weighting process and potentially reduced bias due to confounding. By contrast, NMAs cannot fully adjust for the heterogeneity in patient populations across trials. Moreover, an anchor-based MAIC was employed in line with Signorovitch et al^12,13^ and Phillippo et al.^14^ Compared with an unanchored MAIC (without a common comparator), an anchor-based approach allows for the detection of any unobserved bias caused by remaining effect modifiers and prognostic factors by estimating the relative effect of the “anchor” arms. As a result, the anchored MAIC leads to more informative and rigorous conclusions. In addition, a sensitivity analysis was conducted using data from baseline characteristics of all biologic-experienced patients in the SELECTION and GEMINI 1 trials, which showed that the main findings of this MAIC remained unchanged.

As with all indirect treatment comparisons, this analysis was subject to several limitations. Although the MAIC matched SELECTION trial IPD to comparator populations, there remained differences in the populations compared, especially in the placebo groups, after matching. These differences may be due to unobserved factors, which could not be addressed. Following reweighting of SELECTION patients, the ESS for filgotinib was reduced compared with certain comparator trials (GEMINI 1 [maintenance], OCTAVE 1 and 2, OCTAVE SUSTAIN, UNIFI [maintenance]), which may explain the wide CIs and nonsignificance reported for some comparisons. Owing to the lack of baseline data reported by biologic treatment history, matching in the maintenance phase was conducted in the overall trial populations, whereas our analyses were stratified by biologic treatment history. Furthermore, the definition of corticosteroid-free clinical remission varied between the SELECTION and comparator trials. While efforts were made to harmonize these outcomes with SELECTION trial IPD, the exact definitions in the OCTAVE SUSTAIN and UNIFI trials could not be generated, owing to the absence of data in the SELECTION trial IPD. Therefore, results for the corticosteroid-free clinical remission outcome should be interpreted with caution. Our analyses were not adjusted for multiple comparisons, owing to the lack of guidance for conducting this type of adjustment for indirect treatment comparisons using secondary data. Adjusting for multiple comparisons reduces the number of false positives; however, this increases the probability that a true relation may go unobserved.^35^ Furthermore, the analyses of efficacy outcomes during the maintenance phase had relatively small sample sizes, thereby limiting the statistical power to detect smaller effect sizes. Finally, differences in treatment regimens may exist between clinical trials and real-world practice that may impact the efficacy, safety, and cost-effectiveness of a treatment, which should be considered.

## Conclusions

This analysis of pivotal trials of filgotinib and key comparators indicates that filgotinib is similar in terms of most efficacy outcomes, safety and HRQoL outcomes to current standard of care with vedolizumab, tofacitinib, and ustekinumab. Notably, some potential benefits may exist with the use of filgotinib in terms of clinical response and corticosteroid-free clinical remission compared with vedolizumab IV. This suggests that filgotinib, which has recently been approved for use in patients with UC in the United Kingdom and Europe, may be a valuable oral option for adult patients with moderately to severely active UC.^36^ Real-world studies comparing efficacy and safety are recommended in the future to allow for comparisons to be made in a sufficiently balanced population.

## Supplementary Material

izad037_suppl_Supplementary_Material

## Data Availability

All data relevant to this study are included in the main article or uploaded as supplementary information. Source data may be obtained from previously published material.
