# Supplementary material for: Matching-Adjusted Indirect Comparisons of Filgotinib vs Vedolizumab, Tofacitinib, and Ustekinumab for Moderately to Severely Active Ulcerative Colitis
Source: Inflamm Bowel Dis. 2023 Mar 22;30(1):64–77. doi: 10.1093/ibd/izad037 (PMC10769800; doi:10.1093/ibd/izad037)
Supplement: izad037_suppl_Supplementary_Material [file izad037_suppl_supplementary_material.docx]

# Supplementary Data Content

**Table S1.** Major inclusion criteria across all trials included in the MAIC.

| **Trial** | **SELECTION (filgotinib)** | | **GEMINI 1 (vedolizumab)^17^** | | **VISIBLE 1 (vedolizumab)^19^** | **UNIFI (ustekinumab) ^21^** | | **OCTAVE (tofacitinib)^20^** | | |
| --- | --- | --- | --- | --- | --- | --- | --- | --- | --- | --- |
| **Phase** | **Induction** | **Maintenance** | **Induction** | **Maintenance** | **Maintenance** | **Induction** | **Maintenance** | **Induction**  **OCTAVE  1 & 2** | **Maintenance**  **OCTAVE SUSTAIN** | |
| Demographics | Aged 18–75 years | | Aged 18–80 years | | Aged 18–80 years | Aged ≥18 years | | Aged ≥18 years | | |
| Disease characteristics | For both cohorts A and B: confirmed diagnosis of UC for ≥6 months, based on endoscopy and histopathology, with a minimum disease extent of 15 cm from the anal verge; moderately to severely active UC, determined by a centrally read ES score ≥2, RB score ≥1, SF score ≥1; PGA score ≥2, as determined by the Mayo Clinic scoring system with endoscopy occurring during screening (total score of 6 –12, inclusive); for patients with a history of UC ≥8 years, a surveillance colonoscopy was required prior to screening if one was not performed in the prior 24 months. | | Confirmed diagnosis of UC for ≥6 months; moderately to severely active UC, defined as a TMS of 6–12, with a sigmoidoscopy subscore of ≥2, and disease that extended ≥15 cm from the anal verge; for extensive colitis or pancolitis of >8 years duration or left-sided colitis of >12 years duration, must have documented evidence that surveillance colonoscopy was performed within 12 months of the initial screening visit (may be performed during screening). | | Confirmed diagnosis of UC for ≥6 months, based on histopathology; moderately to severely active UC defined as a TMS of 6–12 (with a centrally read ES score ≥2); evidence of UC extending proximal to the rectum (15 cm of involved colon). | Confirmed diagnosis of UC for ≥3 months; moderately to severely active UC, defined as a TMS of 6–12; subscore of 2 or 3 on the ES component of the Mayo scale, as determined during central review of video endoscopy. | | Confirmed diagnosis of UC for ≥4 months; moderately to severely active disease, which was defined as a TMS of 6–12, with RB score ≥1 and an ES score ≥2. | | |
| Previous treatments | For cohort A (biologic-naive): previous demonstration of inadequate clinical response, loss of response to, or intolerance to at least one of the following agents:   - CS - immunomodulators.   No prior or current use of any anti-TNF agent; no prior or current use of vedolizumab at any time.  For cohort B (biologic-experienced): previous demonstration of an inadequate clinical response, loss of response to, or intolerance of at least one of the following agents:   - anti-TNF agent - vedolizumab.   No use of any anti-TNF agent or vedolizumab ≤8 weeks prior to screening or any other biologic agent ≤8 weeks prior to screening or within 5 times the half-life of the biologic agent prior to screening, whichever was longer. | | Eligible patients were required to have documentation, over the previous 5-year period, of lack of response or unacceptable adverse events with at least one of the following agents:   - glucocorticoids - immunosuppressive medications (azathioprine and 6-mercaptopurine) - anti-TNF agents. | | Eligible patients were required to have inadequate response to, loss of response to, or intolerance to at least one of the following treatments:   - CS - Immunomodulator - anti-TNF agent. | Eligible patients were either required to 1) have had an inadequate response to or unacceptable side effects from anti-TNF agents, vedolizumab, or conventional therapy, or 2) be naïve to biologic therapy or not have demonstrated a history of failure to respond to, or tolerate, a biologic therapy and have a prior or current UC medication history that includes at least one of the following:   - inadequate response to or failure to tolerate current treatment with oral CS or immunomodulators - history of failure to respond to, or tolerate, at least 1 of the following therapies: oral or IV CS or immunomodulators - history of CS dependence. | | Eligible patients must have failed or be intolerant of at least one of the following treatments for UC:   - CS (oral or IV) - azathioprine or 6-mercaptopurine - anti-TNF agents (infliximab or adalimumab) | | |
| Permitted therapies | For both cohorts A and B: oral 5-ASA compounds, azathioprine, 6-mercaptopurine or methotrexate (stable dose for 4 weeks prior to randomization through 10 weeks after randomization); stable dose of oral CS (prednisone prescribed at a stable dose ≤30 mg/day or budesonide prescribed at a stable dose of ≤9 mg/day) for 2 weeks prior to randomization through 14 weeks after randomization. | | Oral 5-ASA compounds (stable dose for the 2 weeks immediately prior to enrollment); oral CS therapy (prednisone at a stable dose ≤30 mg/day, or equivalent steroid) with stable dose for the 4 weeks immediately prior to enrollment if CS have just been initiated, or for the 2 weeks immediately prior to enrollment if CS are being tapered; probiotics with stable dose for the 2 weeks immediately prior to enrollment; antidiarrheals for control of chronic diarrhea; azathioprine or 6-mercaptopurine with stable dose for the 8 weeks immediately prior to enrollment. | | Stable dose of oral mesalamine for the 2 weeks before the first dose of study drug; stable dose of azathioprine for the 8 weeks before first dose of study drug; stable dose of 6-mercaptopurine for the 8 weeks before first dose of study drug; stable dose of oral CS (prednisone <30 mg/d or budesonide <9 mg/d, or equivalent) for the 4 weeks before first dose of study drug if just initiated, or for the 2 weeks prior if being tapered. | Stable doses of aminosalicylates and immunomodulators were maintained from baseline of induction therapy through week 44 of maintenance therapy; oral CS were maintained at a stable dose during the induction trial and tapered when patients entered the maintenance trial. | | Oral aminosalicylates; oral glucocorticoids (at a maximum dose of 25 mg per day of prednisone or a prednisone equivalent); patients receiving non-prohibited concomitant medications for any reason, must be on a stable regimen, which is defined as not starting a new drug or changing dosage with 7 days or 5 half-lives (whichever is longer) prior to first study dose. | | Tapering of glucocorticoids was mandatory. |
| Other | For both cohorts A and B: female patients must be nonpregnant and nonlactating; females of childbearing potential must have had a negative pregnancy test at screening and baseline.  Maintenance Study criteria: completion of Induction Study with MCS response or EBS remission based on Week 10 assessments; willingness to refrain from live or attenuated vaccines during the study and for 12 weeks after last dose; may have received oral CS therapy (prednisone prescribed at a stable dose ≤30 mg/day or budesonide at a dose of ≤9 mg/day) and dose must have remained stable to Week 14. | | Female patients must be post-menopausal for ≥1 year before the screening visit or surgically sterile or, if they are of childbearing potential, agree to practice two effective methods of contraception at the same time (from four weeks before the first dose of study drug through 6 months after the last dose of study drug) or agree to completely abstain from heterosexual intercourse. | | Not reported. | Female patients of childbearing potential must: have a negative serum pregnancy test result at screening and a negative urine pregnancy test result at week 0; agree not to donate eggs for the purposes of assisted reproduction during the study and for 20 weeks after the last study agent administration. | | Female patients of childbearing potential must agree to use a highly effective method of contraception throughout the study and for ≥4 weeks after the last dose of assigned treatment and must have a negative pregnancy test prior to study enrollment; no evidence of active or latent or inadequately treated infection with TB. | | |

Abbreviations: 5-ASA, 5-aminosalicylate; CS, corticosteroid; EBS, endoscopy/bleeding/stool frequency; ES, endoscopy; MCS, components included in the Mayo score; PGA, physician’s global assessment; RB, rectal bleeding; SF, stool frequency; TB, mycobacterium tuberculosis; TMS, Total Mayo Score; UC, ulcerative colitis.

**Table S2.** Major exclusion criteria across all trials included in the MAIC.

| **Trial** | **SELECTION (filgotinib)** | | **GEMINI 1 (Vedolizumab)^17^** | | **VISIBLE 1 (Vedolizumab)^19^** | **UNIFI (Ustekinumab) ^21^** | | **OCTAVE (Tofacitinib)^20^** | |
| --- | --- | --- | --- | --- | --- | --- | --- | --- | --- |
| **Phase** | **Induction** | **Maintenance** | **Induction** | **Maintenance** | **Maintenance** | **Induction** | **Maintenance** | **Induction**  **OCTAVE  1 & 2** | **Maintenance**  **OCTAVE SUSTAIN** |
| Major surgery | For both cohorts A and B: history of major surgery or trauma within 30 days prior to screening; prior surgical intervention for UC (e.g., total colectomy, subtotal colectomy, partial or hemicolectomy, ileostomy, or colostomy) or likely requirement for surgery during the study. | | Patients that had any surgical procedure requiring general anesthesia within 30 days prior to enrollment or were planning to undergo major surgery during the study period were excluded. | | Patients with subtotal or total colectomy. | Within the 2 months before screening, patients that required surgery for active gastrointestinal bleeding, peritonitis, intestinal obstruction, or intra-abdominal or pancreatic abscess requiring surgical drainage, or other conditions possibly confounding the evaluation of benefit from study agent treatment were excluded. | | Patients who had 1) surgery for UC or, in the opinion of the Investigator, were likely to require surgery for UC during the study period or 2) a history of bowel surgery within 6 months prior to baseline were excluded or 3) significant trauma or major surgery within 4 weeks of screening visit were excluded. | |
| Non-biologic therapy | For both cohorts A and B: active clinically significant infection, or any infection requiring hospitalization or treatment with IV anti-infectives within 30 days of screening (or 8 weeks of Day 1), or any infection requiring oral anti-infective therapy within 2 weeks of screening (or 6 weeks of Day 1); history of treatment with lymphocyte-depleting therapies (e.g., cyclophosphamide, total lymphoid irradiation), prohibited any time before the study; on any chronic systemic (oral or IV) anti-infective therapy for chronic infection (e.g., pneumocystis, cytomegalovirus, HZ, atypical mycobacteria); use of prohibited concomitant medications including:   - strong P-gp inducers (anticonvulsants, antimycobacterials, herbal/natural supplements) within 30 days prior to screening through the end of study - IBD medications   - antidiarrheal agents (ie, loperamide and diphenoxylate/atropine) within 2 weeks prior to screening through the end of study   - cyclosporine, thalidomide, tacrolimus, leflunomide, and any investigational agent within 30 days prior to screening through the end of study   - any JAK inhibitor, any time before and through the end of the study   - chronic NSAIDs (ie, aspirin, ibuprofen, naproxen, diclofenac, indomethacin, COX-2 inhibitors), from screening through the end of the study. | | Patients treated, within 30 days prior to enrollment, with any of the following were excluded:   - - a non-biologic investigational therapy   - an approved non-biologic therapy in an investigational protocol   - non-biologic therapies (e.g., cyclosporine, thalidomide)   - chronic use of NSAID. | | Patients who were treated with any of the following nonbiologic therapies within 30 days or 5 half-lives of screening (whichever was longer) were excluded:   - - cyclosporine   - tacrolimus   - thalidomide   - methotrexate   - tofacitinib. | Patients treated with any of the following were excluded:   - - an investigational drug within 4 weeks before first administration of study agent or within 5 half-lives of the investigational agent, whichever is longer   - agents that deplete B or T cells (e.g., rituximab, alemtuzumab) within 12 months of first study agent administration or continue to manifest depletion of B or T cells more than 12 months after completion of therapy with lymphocyte-depleting agents   - apheresis (eg, Adacolumn or Cellsorba apheresis) within 2 weeks before the first administration of study agent   - interleukin-12 or interleukin-23 antagonists   - other conventional therapies within 2 to 4 weeks of trial entry. | | Patients treated with any of the following were excluded:   - azathioprine, 6-mercaptopurine or methotrexate within 2 weeks prior to baseline - cyclosporine, mycophenolate or tacrolimus within 4 weeks prior to baseline - previously receiving leukocyte apheresis - interferon therapy within 8 weeks prior to baseline - IV CS within 2 weeks prior to baseline - prohibited concomitant medications, including moderate to potent CYP3A inducers or inhibitors in the specified time periods prior to the first dose of study drug or were expected to receive any of these medications during the study period. | |
| Biologic therapy | For both cohorts A and B: history of treatment with lymphocyte-depleting therapies, including but not limited to alemtuzumab and rituximab, prohibited any time before the study; integrin antagonist (ie, vedolizumab and natalizumab) 8 weeks prior to screening; interleukin antagonist ustekinumab 12 weeks prior to screening; any investigational biologic agent within 8 weeks prior to screening (or at least 5 half-lives); antibody-based or other systemic biologics, eg, denosumab, trastuzumab (allowed upon approval of the medical monitor).  For cohort A: prior use of any anti-TNF agent including (but not limited to) infliximab, adalimumab, golimumab, certolizumab, or biosimilar agents at any time; prior or current use of vedolizumab at any time.  For cohort B: use of any anti-TNF agent or vedolizumab within ≤8 weeks prior to screening, or any other biologic agent ≤8 weeks prior to screening or within 5 half-lives of the biologic agent prior to screening, whichever was longer. | | History of treatment with anti-TNF agents within 60 days before enrollment; previously treated with vedolizumab, natalizumab, efalizumab or rituximab. | | Prior exposure to any anti-integrin therapies (e.g., vedolizumab, natalizumab, efalizumab, etrolizumab, AMG 181), anti-MAdCAM-1 antibodies or rituximab; exposure to any biologics within 60 days or 5 half-lives of screening (whichever was longer). | History of treatment with anti-TNF agents within 8 weeks before trial entry or vedolizumab within 4 months before trial entry; a biologic therapy targeted at IL-12 and/or IL-23 (e.g., ustekinumab, briakinumab, guselkumab); natalizumab within 12 months of administration of first study agent; vedolizumab within 4 months before first administration of study agent. | | History of treatment with anti-TNF agents (e.g., infliximab, adalimumab or certolizumab) within 8 weeks prior to baseline; anti-adhesion molecule therapy taken within 1 year (e.g., natalizumab or any investigational anti-adhesion molecule therapy); previous participation in any study of tofacitinib. | |
| Topical (rectal) treatment | For both cohorts A and B: use of rectal formulations of 5-ASA compounds or rectal CS 2 weeks prior to screening; use of CS 30 days prior to screening. | | Topical (rectal) treatment with 5-ASA or CS enemas/suppositories within 2 weeks of the administration of the first dose of study drug. | | Not reported. | Rectal CS within 2 weeks before first administration of study drug; rectal 5-ASA compounds within 2 weeks before first administration of study drug. | | Rectal CS within 2 weeks prior to baseline; rectal 5-ASA within 2 weeks prior to baseline. | |
| Existing/prior conditions | For both cohorts A and B: exhibited acute severe UC as defined by ≥6 bloody stools daily and 1 or more of the following:   - body temperature ≥100.4°F (or 38°C) - pulse >90 beats per minute.   Known hypersensitivity to filgotinib, its metabolites or formulation excipients; presence of Crohn’s disease, indeterminate colitis, ischemic colitis, fulminant colitis, isolated ulcerative proctitis or toxic mega-colon; history or evidence of incompletely resected colonic mucosal dysplasia; stool sample positive for Clostridium difficile toxin, pathogenic Escherichia coli, Salmonella species, Shigella species, Campylobacter species or Yersinia species; stool sample positive for ova and parasites test, unless approved by the medical monitor; infection with HIV, HBV or HCV; presence of Child-Pugh Class C hepatic impairment; untreated active TB or history of latent TB; history of malignancy in the last 5 years except for patients who had been successfully treated for non-melanoma skin cancer or cervical carcinoma in situ; history of lymphoproliferative disorder, lymphoma, leukemia, myeloproliferative disorder or multiple myeloma; history of cytapheresis ≤2 months prior to screening; any chronic medical condition (including, but not limited to, cardiac or pulmonary disease, or substance abuse) or psychiatric problem that, in the opinion of the investigator or sponsor, would have made the patient unsuitable for the study or would have prevented compliance with the study protocol procedures; history of opportunistic infection or immunodeficiency syndrome; history of disseminated Staphylococcus aureus; history of symptomatic HZ or herpes simplex within 12 weeks of screening, or any history of disseminated herpes simplex, disseminated HZ, ophthalmic zoster, or central nervous system zoster. | | Diagnosis of Crohn’s colitis or indeterminate colitis; history or evidence of adenomatous colonic polyps that have not been removed; toxic megacolon; abdominal abscess; symptomatic colonic stricture, stoma or a history of colectomy; colonic dysplasia or adenomas, and malignant neoplasms; evidence of or treatment for Clostridium difficile infection within 60 days or other intestinal pathogen within 30 days prior to enrollment; any unstable or uncontrolled cardiovascular, pulmonary, hepatic, renal, gastrointestinal, genitourinary, hematological, coagulation, immunological, endocrine/metabolic that would confound the study results; an increased risk of infectious complications (e.g., as a result of recent pyogenic infection, enteric pathogens detected on stool analysis, active or latent TB, HIV, HBV or HCV, or recent live vaccination); clinically significant extra-intestinal infection (eg, pneumonia, pyelonephritis) within 30 days prior to enrollment; any identified congenital or acquired immunodeficiency; any history of malignancy, except for the following: (a) adequately-treated non-metastatic basal cell skin cancer, (b) any other type of non-melanoma skin cancer that had been adequately treated and had not recurred for ≥1 year prior to enrollment and (c) adequately treated in situ cervical cancer that had not recurred for ≥1 year prior to enrollment; history of any major neurological disorders, including stroke, multiple sclerosis, brain tumor or neurodegenerative disease. | | Suspected or confirmed diagnosis of Crohn's entercolitis, indeterminate colitis, ischemic colitis, radiation colitis, diverticular disease associated with colitis or microscopic colitis; evidence of abdominal abscess or toxic megacolon; history or evidence of unresected adenomatous colonic polyps or colonic mucosal dysplasia; ileostomy, colostomy or known fixed symptomatic stenosis of the intestine; chronic HBV infection, chronic HCV infection, or infected with HIV or TB (active or latent); history of any major neurological disorders, including stroke, multiple sclerosis, brain tumor, demyelinating or neurodegenerative disease. | Severe extensive colitis as evidenced by 1) current hospitalization for the treatment of UC or 2) investigator judgment that the patient is likely to require a colectomy within 12 weeks of baseline or 3) symptom complex at screening or baseline visits that includes ≥4 of the following:   - diarrhea with ≥6 bowel movements/day with macroscopic blood in stool - focal severe or rebound abdominal tenderness - persistent fever (≥37.5°C) - tachycardia (>90 beats/minute) - anemia (haemoglobin <8.5 g/dL).   UC limited to the rectum only or to <20 cm of the colon; diagnosis of indeterminate colitis, microscopic colitis, ischemic colitis, or Crohn’s disease or clinical findings suggestive of Crohn’s disease; stool culture or other examination positive for an enteric pathogen, including Clostridium difficile toxin, in the previous 4 months, unless a repeat examination is negative and there are no signs of ongoing infection with that pathogen; imminent colectomy; gastrointestinal conditions that would result in surgery or confound disease-activity assessment; presence of stoma; history of colonic mucosal dysplasia; presence on screening endoscopy of adenomatous colonic polyps; active infections (including tuberculosis); any known malignancy or had a history of malignancy (with the exception of basal cell carcinoma; squamous cell carcinoma in situ of the skin; or cervical carcinoma in situ that had been treated with no evidence of recurrence; or squamous cell carcinoma of the skin that had been treated with no evidence of recurrence within 5 years before screening); transplanted organ (with the exception of a corneal transplant performed >12 weeks before screening); previously undergone allergy immunotherapy for prevention of anaphylactic reactions. | | Patients without previous treatment for UC were excluded; the presence of clinical findings suggestive of Crohn’s disease; positive stool examinations for enteric pathogens, pathogenic ova or parasites, or Clostridium difficile toxin at screening; UC limited to the distal 15 cm of colon; clinical signs of fulminant colitis, toxic megacolon, or indeterminate, microscopic, ischemic, adenomatous polyps, or infectious colitis; evidence of colonic adenomas or dysplasia; current or recent history of severe, progressive, or uncontrolled renal, hepatic, hematological, gastrointestinal, metabolic (including uncontrolled hypercholesterolemia), endocrine, pulmonary, cardiac, neurological disease; clinically significant infections currently or within 6 months of baseline, a history of any infection requiring antimicrobial therapy within 2 weeks of baseline, or a history of any infection otherwise judged by the investigator to have the potential for exacerbation by participation in the study; a history of more than one episode of HZ, a history of disseminated HZ or disseminated herpes simplex; infected with HIV or HBV or HCV; history of any lymphoproliferative disorder, history of lymphoma, leukemia, myeloproliferative disorders, multiple myeloma, or signs and symptoms suggestive of current lymphatic disease; malignancies or a history of malignancies, with the exception of adequately treated or excised non-metastatic basal cell or squamous cell cancer of the skin; evidence of or suspected liver disease; patients at risk for colorectal cancer must have had a colonoscopy. | |
| Other | For both cohorts A and B: administration of a live or attenuated vaccine within 30 days of randomization; laboratory values outside of the following:   - ALT and AST concentrations must be within 2 times the ULN for the laboratory conducting the test - estimated CrCl ≥40 mL/min - platelets ≥100 × 10^9^/L - haemoglobin ≥8.0 g/dL - neutrophils ≥1.5 × 10^3^/μL - WBC count ≥2.5 × 10^3^/L - lymphocyte count <750/mm^3^   Maintenance Study criterion: use of prohibited concomitant medications as listed above. | | Pregnancy or lactation; any live vaccinations within 30 days prior to study drug administration except for the influenza vaccine; history of alcohol dependence or illicit drug use, within 1 year before enrollment; the following laboratory values at screening:   - - serum creatinine >2 times the ULN   - ALT or AST >3 times the ULN   - platelet count <100 × 10^9^/L or >1200 × 10^9^/L   - Hemoglobin level <8 g/dL   - WBC count <3 × 10^9^/L   - Lymphocyte count <0.5 × 10^9^/L   - Alkaline phosphatase >3 × ULN. | | Not reported. | History of drug or alcohol abuse according to the Diagnostic and Statistical Manual of Mental Disorders 4th edition (DSM-IV), within 1 year before screening; currently participating or intends to participate in any other study using an investigational agent or procedure during participation in this study; laboratory values outside of the following:   - - serum creatinine <1.5 mg/dL   - ALT and AST concentrations must be within 2 times the ULN for the laboratory conducting the test   - platelets ≥100 × 10^3^/μL   - haemoglobin ≥8.0 g/dL   - neutrophils ≥1.5 × 10^3^/μL   - WBC count ≥2.5 × 10^3^/μL. | | Screening 12-lead ECG that demonstrates clinically relevant abnormalities which may affect patient safety or interpretation of study results; patients who have been vaccinated with live or attenuated vaccine within 6 weeks of baseline or scheduled to receive these vaccines during study period or within 6 weeks after last dose of study medication; patients with the following laboratory values at screening:   - - total bilirubin, AST or ALT >1.5 times the ULN   - thrombocytopenia, as defined by a platelet count <100 x 10^^9^/L (<100,000/mm^3^)   - haemoglobin levels <9.0 g/dL   - an absolute WBC count of <3.0 x 10^9^/L (<3000/mm^3^) or absolute neutrophil count of <1.2 x 10^9^/L (<1200/mm^3^) or absolute lymphocyte count of <0.5 x 10^9^/L (<500/mm^3^) (or <0.75 x 10^9^/L (<750/mm^3^) in the UK)   - estimated GFR <40 mL/min based on Cockcroft-Gault calculation. | |

Abbreviations: 5-ASA, 5-aminosalicylate; ALT, alanine aminotransferase; AST, aspartate aminotransferase; CS, corticosteroid; EBS, endoscopy/bleeding/stool frequency; ES, endoscopy; HBV, hepatitis B virus; HCV, hepatitis C virus; HIV, human immunodeficiency virus; HZ, herpes zoster; IBD, Inflammatory bowel disease; MCS, components included in the Mayo score; NSAID, nonsteroidal anti-inflammatory drug; PGA, physician’s global assessment; RB, rectal bleeding; SF, stool frequency; TB, tuberculosis; TMS, Total Mayo Score; UC, ulcerative colitis; ULN, upper limit of the normal range.

**Table S3.** Baseline characteristics of the biologic-naive induction population in the SELECTION (filgotinib) and GEMINI 1 (vedolizumab IV) trials, before and after matching.

| **Baseline matching variables** | **Before matching** | | | | | | **After matching** | | | |
| --- | --- | --- | --- | --- | --- | --- | --- | --- | --- | --- |
|  | **SELECTION** | | **GEMINI 1** | |  | | **SELECTION** | | **GEMINI 1** | |
|  | **A. Filgotinib**  **(n = 245)** | **B. Placebo**  **(n = 137)** | **C. Vedolizumab IV**  **(n = 130)** | **D. Placebo**  **(n = 76)** | ***P* value^a^**  **A vs C^a^** | ***P* value^a^**  **B vs D^a^** | **Filgotinib**  **(n = 245,**  **ESS = 167)** | **Placebo**  **(n = 137,**  **ESS = 92)** | **Vedolizumab IV**  **(n = 130)** | **Placebo**  **(n = 76)** |
| **Male** | 123 (50.2%) | 87 (63.5%) | 69 (53.1%) | 47 (61.8%) | .67 | .93 | 53.1% | 61.8% | 53.1% | 61.8% |
| **Age, y** | 42.3 ± 13.1 | 41.3 ± 12.9 | 39.7 ± 13.1 | 40.5 ± 11.7 | .07 | .65 | 39.7 ± 12.8 | 40.5 ± 12.6 | 39.7 ± 13.1 | 40.5 ± 11.7 |
| **Current smoker** | 15 (6.1%) | 5 (3.6%) | 7 (5.4%) | 7 (9.2%) | .95 | .17 | 5.4% | 9.2% | 5.4% | 9.2% |
| **Weight, kg** | 70.1 ± 17.9 | 69.5 ± 15.9 | 69.2 ± 16.6 | 70.0 ± 18.8 | .64 | .83 | 69.2 ± 17.7 | 70.0 ± 16.0 | 69.2 ± 16.6 | 70.0 ± 18.8 |
| **Disease duration, y** | 7.2 ± 6.9 | 6.4 ± 7.4 | 5.8 ± 5.2 | 6.1 ± 6.4 | **<.05^b^** | .74 | 5.8 ± 6.0 | 6.1 ± 7.0 | 5.8 ± 5.2 | 6.1 ± 6.4 |
| **Total Mayo score at induction baseline** | 8.6 ± 1.3 | 8.7 ± 1.3 | 8.4 ± 1.8 | 8.5 ± 1.5 | .24 | .37 | 8.4 ± 1.3 | 8.5 ± 1.3 | 8.4 ± 1.8 | 8.5 ± 1.5 |
| **Concomitant corticosteroid use at induction baseline** | 74 (30.2%) | 42 (30.7%) | 73 (56.2%) | 44 (57.9%) | **<.001^c^** | **<.001^c^** | 56.2% | 57.9% | 56.2% | 57.9% |

Values are n (%), %, or mean ± SD, unless otherwise indicated.

Abbreviations: ESS, effective sample size; IV, intravenous.

^a^*P* values for continuous variables were calculated using the Wald test. *P* values for categorical variables were calculated using the χ^2^ test. The Fisher exact test was used for categorical variables with small frequency (ie, n < 5). ^b^*P* value <.05. ^c^*P* value <.001.

**Table S4.** Baseline characteristics of the biologic-naive induction population in the SELECTION (filgotinib) and OCTAVE 1 and 2 (tofacitinib) trials, before and after matching.

| **Baseline matching variables** | **Before matching** | | | | | | **After matching** | | | |
| --- | --- | --- | --- | --- | --- | --- | --- | --- | --- | --- |
|  | **SELECTION** | | **OCTAVE 1 and 2** | |  | | **SELECTION** | | **OCTAVE 1 and 2** | |
|  | **A. Filgotinib**  **(n = 245)** | **B. Placebo**  **(n = 137)** | **C. Tofacitinib**  **(n = 417)** | **D. Placebo**  **(n = 104)** | ***P* value^a^**  **A vs C^a^** | ***P* value^a^**  **B vs D^a^** | **Filgotinib**  **(n = 245,**  **ESS = 213)** | **Placebo**  **(n = 137,**  **ESS = 120)** | **Tofacitinib**  **(n = 417)** | **Placebo**  **(n = 104)** |
| **Male** | 123 (50.2%) | 87 (63.5%) | 249 (59.7%) | 64 (61.5%) | **<.05^b^** | .86 | 59.7% | 61.5% | 59.7% | 61.5% |
| **Age, y** | 42.3 ± 13.1 | 41.3 ± 12.9 | 41.1 ± 13.5 | 43.2 ± 13.9 | .25 | .27 | 41.1 ± 12.8 | 43.2 ± 12.9 | 41.1 ± 13.5 | 43.2 ± 13.9 |
| **Never smoked** | 175 (71.4%) | 110 (80.3%) | 282 (67.6%) | 75 (72.1%) | .35 | .18 | 67.6% | 72.1% | 67.6% | 72.1% |
| **Total Mayo score at induction baseline** | 8.6 ± 1.3 | 8.7 ± 1.3 | 8.8 ± 1.4 | 8.8 ± 1.4 | .08 | .52 | 8.8 ± 1.3 | 8.8 ± 1.4 | 8.8 ± 1.4 | 8.8 ± 1.4 |
| **Concomitant corticosteroid use at induction baseline** | 74 (30.2%) | 42 (30.7%) | 173 (41.5%) | 44 (42.3%) | **<.01^c^** | .08 | 41.5% | 42.3% | 41.5% | 42.3% |

Values are n (%), %, or mean ± SD, unless otherwise indicated.

Abbreviations: ESS, effective sample size; SD, standard deviation; TNF, tumor necrosis factor.

^a^*P* values for continuous variables were calculated using the Wald test. *P* values for categorical variables were calculated using the χ^2^ test. The Fisher exact test was used for categorical variables with small frequency (ie, n < 5). ^b^*P* value <.05. ^c^*P* value <.01.

**Table S5.** Baseline characteristics of the biologic-experienced induction population in the SELECTION (filgotinib) and GEMINI 1 (vedolizumab IV; TNF failure only) trials, before and after matching.

| **Baseline matching variables** | **Before matching** | | | | | | **After matching** | | | |
| --- | --- | --- | --- | --- | --- | --- | --- | --- | --- | --- |
|  | **SELECTION** | | **GEMINI 1** | |  | | **SELECTION** | | **GEMINI 1** | |
|  | **A. Filgotinib**  **(n = 218)** | **B. Placebo**  **(n = 120)** | **C. Vedolizumab IV**  **(n = 82)** | **D. Placebo**  **(n = 63)** | ***P* value^a^**  **A vs C^a^** | ***P* value^a^**  **B vs D^a^** | **Filgotinib**  **(n = 218,**  **ESS = 145)** | **Placebo**  **(n = 120,**  **ESS = 77)** | **Vedolizumab IV**  **(n = 82)** | **Placebo**  **(n = 63)** |
| **Male** | 128 (58.7%) | 72 (60.0%) | 50 (61.0%) | 35 (55.6%) | .82 | .67 | 61.0% | 55.6% | 61.0% | 55.6% |
| **Age, y** | 43.2 ± 13.9 | 43.9 ± 15.5 | 39.7 ± 12.5 | 41.8 ± 13.1 | **<.05^b^** | .34 | 39.7 ± 13.5 | 41.8 ± 15.4 | 39.7 ± 12.5 | 41.8 ± 13.1 |
| **Current smoker** | 7 (3.2%) | 4 (3.3%) | 4 (4.9%) | 1 (1.6%) | .50 | .66 | 4.9% | 1.6% | 4.9% | 1.6% |
| **Weight, kg** | 72.7 ± 17.9 | 73.1 ± 17.0 | 74.9 ± 17.0 | 74.2 ± 16.4 | .33 | .67 | 74.9 ± 18.9 | 74.2 ± 17.2 | 74.9 ± 17.0 | 74.2 ± 16.4 |
| **Disease duration, y** | 9.6 ± 7.1 | 9.8 ± 8.4 | 6.4 ± 5.0 | 8.0 ± 7.6 | **<.001^c^** | .14 | 6.4 ± 4.9 | 8.0 ± 6.4 | 6.4 ± 5.0 | 8.0 ± 7.6 |
| **Total Mayo score at induction baseline** | 9.3 ± 1.4 | 9.4 ± 1.4 | 8.7 ± 1.8 | 8.6 ± 1.9 | **<.05^b^** | **<.01^d^** | 8.7 ± 1.5 | 8.6 ± 1.4 | 8.7 ± 1.8 | 8.6 ± 1.9 |
| **Concomitant corticosteroid use at induction baseline** | 107 (49.1%) | 57 (47.5%) | 43 (52.4%) | 35 (55.6%) | .70 | .38 | 52.4% | 55.6% | 52.4% | 55.6% |

Values are n (%), %, or mean ± SD, unless otherwise indicated.

Abbreviations: ESS, effective sample size; IV, intravenous.

^a^*P* values for continuous variables were calculated using the Wald test. *P* values for categorical variables were calculated using the χ^2^ test. The Fisher exact test was used for categorical variables with small frequency (ie, n < 5). ^b^*P* value <.05. ^c^*P* value <.001. ^d^*P* value <.01.

**Table S6.** Baseline characteristics of the biologic-experienced induction population in the SELECTION (filgotinib) and OCTAVE 1 and 2 (tofacitinib) trials, before and after matching.

| **Baseline matching variables** | **Before matching** | | | | | | **After matching** | | | |
| --- | --- | --- | --- | --- | --- | --- | --- | --- | --- | --- |
|  | **SELECTION** | | **OCTAVE 1 and 2** | |  | | **SELECTION** | | **OCTAVE 1 and 2** | |
|  | **A. Filgotinib**  **(n = 262)** | **B. Placebo**  **(n = 142)** | **C. Tofacitinib**  **(n = 488)** | **D. Placebo**  **(n = 130)** | ***P* value^a^**  **A vs C^a^** | ***P* value^a^**  **B vs D^a^** | **Filgotinib**  **(n = 262,**  **ESS = 208)** | **Placebo**  **(n = 142,**  **ESS = 110)** | **Tofacitinib**  **(n = 488)** | **Placebo**  **(n = 130)** |
| **Male** | 148 (56.5%) | 86 (60.6%) | 287 (58.8%) | 68 (52.3%) | .59 | .21 | 58.8% | 52.3% | 58.8% | 52.3% |
| **Age, y** | 43.3 ± 14.2 | 44.4 ± 14.9 | 41.3 ± 14.1 | 39.4 ± 14.5 | .07 | **<.01^b^** | 41.3 ± 13.9 | 39.4 ± 14.9 | 41.3 ± 14.1 | 39.4 ± 14.5 |
| **Never smoked** | 181 (69.1%) | 94 (66.2%) | 287 (58.8%) | 86 (66.2%) | **<.01^b^** | 1.00 | 58.8% | 66.2% | 58.8% | 66.2% |
| **Total Mayo score at induction baseline** | 9.2 ± 1.4 | 9.3 ± 1.4 | 9.1 ± 1.4 | 9.1 ± 1.5 | .20 | .31 | 9.1 ± 1.4 | 9.1 ± 1.4 | 9.1 ± 1.4 | 9.1 ± 1.5 |
| **Concomitant corticosteroid use at induction baseline** | 122 (46.6%) | 62 (43.7%) | 239 (49.0%) | 69 (53.1%) | .58 | .15 | 49.0% | 53.1% | 49.0% | 53.1% |
| **History of anti-TNF failure at induction baseline** | 218 (83.2%) | 120 (84.5%) | 465 (95.3%) | 124 (95.4%) | **<.001^c^** | **<.01^b^** | 95.3% | 95.4% | 95.3% | 95.4% |

Values are n (%), %, or mean ± SD, unless otherwise indicated.

Abbreviations: ESS, effective sample size; TNF, tumor necrosis factor.

^a^*P* values for continuous variables were calculated using the Wald test. *P* values for categorical variables were calculated using the χ^2^ test. The Fisher exact test was used for categorical variables with small frequency (ie, n < 5). ^b^*P* value <.01. ^c^*P* value <.001.

**Table S7.** Baseline characteristics of the overall induction population in the SELECTION (filgotinib) and UNIFI (ustekinumab) trials, before and after matching.

| **Baseline matching variables** | **Before matching** | | | | | | **After matching** | | | |
| --- | --- | --- | --- | --- | --- | --- | --- | --- | --- | --- |
|  | **SELECTION** | | **UNIFI** | |  | | **SELECTION** | | **UNIFI** | |
|  | **A. Filgotinib**  **(n = 507)** | **B. Placebo**  **(n = 279)** | **C. Ustekinumab**  **(n = 322)** | **D. Placebo**  **(n = 319)** | ***P* value**  **A vs C^a^** | ***P* value**  **B vs D^a^** | **Filgotinib**  **(n = 507,**  **ESS = 454)** | **Placebo**  **(n = 279,**  **ESS = 252)** | **Ustekinumab**  **(n = 322)** | **Placebo**  **(n = 319)** |
| **Male** | 271 (53.5%) | 173 (62.0%) | 195 (60.6%) | 197 (61.8%) | .05 | 1.00 | 60.6% | 61.8% | 60.6% | 61.8% |
| **Age, y** | 42.8 ± 13.7 | 42.9 ± 14.0 | 41.7 ± 13.7 | 41.2 ± 13.5 | .26 | .14 | 41.7 ± 13.6 | 41.2 ± 13.8 | 41.7 ± 13.7 | 41.2 ± 13.5 |
| **Weight, kg** | 71.6 ± 18.3 | 71.3 ± 16.4 | 73.0 ± 19.3 | 72.9 ± 16.8 | .31 | .25 | 73.0 ± 18.6 | 72.9 ± 17.4 | 73.0 ± 17.3 | 72.9 ± 16.8 |
| **Disease duration, y** | 8.5 ± 7.4 | 8.3 ± 8.0 | 8.2 ± 7.8 | 8.0 ± 7.2 | .53 | .61 | 8.2 ± 7.2 | 8.0 ± 7.7 | 8.2 ± 7.8 | 8.0 ± 7.2 |
| **Total Mayo score at induction baseline** | 8.9 ± 1.4 | 9.0 ± 1.4 | 8.9 ± 1.5 | 8.9 ± 1.6 | .75 | .47 | 8.9 ± 1.4 | 8.9 ± 1.4 | 8.9 ± 1.5 | 8.9 ± 1.6 |
| **Concomitant corticosteroid use at induction baseline** | 196 (38.7%) | 104 (37.3%) | 168 (52.2%) | 157 (49.2%) | **<.001^b^** | **<.01^c^** | 52.2% | 49.2% | 52.2% | 49.2% |
| **History of biologic failure at induction baseline** | 246 (48.5%) | 132 (47.3%) | 166 (51.6%) | 161 (50.5%) | .44 | .49 | 51.6% | 50.5% | 51.6% | 50.5% |

Values are n (%), %, or mean ± SD, unless otherwise indicated.

Abbreviations: ESS, effective sample size.

^a^*P* values for continuous variables were calculated using the Wald test. *P* values for categorical variables were calculated using the χ^2^ test. The Fisher exact test was used for categorical variables with small frequency (ie, n < 5). ^b^*P* value <.001. ^c^*P* value <.01.

**Table S8.** Definitions of outcomes compared in the MAIC for the (A) induction and (B) maintenance periods.

A

| **Clinical trial(s)** | **Clinical remission** | **Endoscopic improvement** | **Clinical response** |
| --- | --- | --- | --- |
| SELECTION | SELECTION IPD were used to match the definition used in the comparator trial | SELECTION IPD were used to match the definition used in the comparator trial | SELECTION IPD were used to match the definition used in the comparator trial |
| GEMINI 1 | Patients with TMS ≤2 and no subscore >1 | Patients with endoscopic subscore ≤1 | Patients with a reduction from baseline in TMS of ≥3 points and ≥30%, and reduction from baseline in the rectal bleeding subscore ≥1 or absolute rectal bleeding subscore ≤1 |
| OCTAVE 1 and 2 | Patients with TMS ≤2 and no subscore >1, and rectal bleeding subscore of 0 |  |  |
| UNIFI | Patients with TMS ≤2 and no subscore >1 |  |  |

B

| **Clinical trial(s)** | **Clinical remission** | **Endoscopic improvement** | **Clinical response** | **Sustained clinical remission** | **Corticosteroid-free clinical remission** |
| --- | --- | --- | --- | --- | --- |
| SELECTION | SELECTION IPD were used to match the definition used in the comparator trial | SELECTION IPD were used to match the definition used in the comparator trial | SELECTION IPD were used to match the definition used in the comparator trial | SELECTION IPD were used to match the definition used in the comparator trial | SELECTION IPD were used to match the definition used in the comparator trial to the best extent |
| GEMINI 1, VISIBLE 1 | Patients with TMS ≤2 and no subscore >1 | Patients with endoscopic subscore ≤1 | Not assessed in MAIC | Patients with clinical remission at the end of both the induction and maintenance phase | Clinical remission with no corticosteroid use at the end of maintenance among patients who were on corticosteroids at induction baseline^a^ |
| OCTAVE SUSTAIN | Patients with TMS ≤2, no subscore >1, and rectal bleeding subscore of 0 |  | Patients with a reduction from induction baseline in TMS of ≥3 points and ≥30%, and reduction from induction baseline in the rectal bleeding subscore ≥1  or absolute rectal bleeding subscore ≤1 | Not assessed in MAIC | Clinical remission (rectal bleeding score of 0) with no corticosteroid use for at least 4 weeks before the end of maintenance among patients who were on corticosteroids at re-baseline^b^ |
| UNIFI | Patients with TMS ≤2 and no subscore >1 |  |  | Patients with clinical remission at the end of maintenance phase among those in remission at re-baseline | Clinical remission with no corticosteroid use at the end of maintenance among all patients at re-baseline^c^ |

Abbreviations: IPD, individual patient data; MAIC, matching-adjusted indirect comparison; TMS, total Mayo score.

^a^SELECTION IPD were used to fully match the definitions of corticosteroid-free clinical remission used in GEMINI 1 and VISIBLE 1.

^b^In the comparison with OCTAVE, the ‘4 weeks’ requirement could not be met with SELECTION IPD. Therefore, the definition of corticosteroid-free clinical remission used in the MAIC was defined as clinical remission with no corticosteroid use at the end of maintenance among patients who were on corticosteroids at re-baseline (maintenance baseline).

^c^In the comparison with UNIFI, SELECTION IPD were used to match the definition of corticosteroid-free clinical remission in UNIFI with an assumption. In SELECTION, the use of corticosteroids at the end of maintenance was unknown among those who did not take corticosteroids at re-baseline (maintenance baseline). The definition used in the MAIC assumed that patients who did not take corticosteroids at re-baseline were not taking corticosteroids at the end of maintenance.

## Appendix 1: Comparative Efficacy Between Filgotinib and Each Comparator in the Induction Phase

### Filgotinib vs Vedolizumab

Differences between filgotinib 200 mg and intravenous (IV) vedolizumab 300 mg were similar after matching for all outcomes among the biologic-naive induction subgroup (**Table S9**).

Filgotinib 200 mg was associated with a higher clinical response rate compared with vedolizumab IV 300 mg (odds ratio [OR], 2.4; 95% confidence interval [CI], 1.0 to 5.5; *P* < .05) among the biologic-experienced induction population. No significant difference was observed between filgotinib 200 mg and vedolizumab IV 300 mg in clinical remission or endoscopic improvement among the biologic-experienced induction population.

### Filgotinib vs Tofacitinib

Differences between filgotinib 200 mg and tofacitinib 10 mg were similar after matching for all outcomes among the biologic-naive induction population (**Table S10**).

Filgotinib 200 mg was associated with a higher clinical response rate compared with tofacitinib 10 mg (OR, 2.0; 95% CI, 1.1 to 3.7; *P* < .05) among the biologic-experienced induction population. However, a significant difference was also observed between the placebo groups in SELECTION and OCTAVE 1 and 2 (OR, 0.5; 95% CI, 0.3 to 0.9; *P* < .05). There was no significant difference observed between filgotinib 200 mg and tofacitinib 10 mg in clinical remission or endoscopic improvement among the biologic-experienced induction population.

### Filgotinib vs Ustekinumab

No significant difference was observed between filgotinib 200 mg and ustekinumab 6 mg/kg in any outcomes in either the biologic-naive or the biologic-experienced induction subgroups (**Table S11**).

**Table S9.** Odds ratio between filgotinib 200 mg and vedolizumab IV 300 mg on efficacy outcomes in the (A) biologic-naive and (B) biologic‑experienced (anti-TNF failure only) subgroups during the induction period.

A

| **Filgotinib 200 mg vs vedolizumab IV** | **Before matching** | | | **After matching** | | |
| --- | --- | --- | --- | --- | --- | --- |
|  | **OR** | **95% CI** | ***P* value^a^** | **OR** | **95% CI** | ***P* value^a^** |
| **Clinical remission^b^** | 0.5 | (0.2 to 1.5) | .24 | 0.6 | (0.2 to 1.7) | .32 |
| **Endoscopic improvement^c^** | 0.7 | (0.4 to 1.3) | .23 | 0.7 | (0.4 to 1.5) | .39 |
| **Clinical response^d^** | 0.7 | (0.4 to 1.3) | .24 | 0.8 | (0.4 to 1.4) | .37 |

B

| **Filgotinib 200 mg vs vedolizumab IV** | **Before matching** | | | **After matching** | | |
| --- | --- | --- | --- | --- | --- | --- |
|  | **OR** | **95% CI** | ***P* value^a^** | **OR** | **95% CI** | ***P* value^a^** |
| **Clinical remission^b^** | 0.8 | (0.1 to 5.3) | .85 | 0.7 | (0.1 to 4.9) | .74 |
| **Endoscopic improvement^c^** | 1.9 | (0.7 to 5.1) | .21 | 1.5 | (0.5 to 4.1) | .48 |
| **Clinical response^d^** | 3.0 | (1.4 to 6.5) | **<.01^e^** | 2.4 | (1.0 to 5.5) | **<.05^f^** |

Abbreviations: CI, confidence interval; IV, intravenous; OR, odds ratio.

^a^*P* values were calculated using the Wald test. ^b^Clinical remission is defined as the proportion of patients with total Mayo score ≤2 and no subscore >1 at the end of induction. ^c^Endoscopic improvement is defined as the proportion of patients with endoscopic subscore ≤1 at the end of induction. ^d^Clinical response is defined as the proportion of patients with a reduction from baseline in total Mayo score ≥3 and ≥30%, and reduction from baseline in the rectal bleeding subscore ≥1 or an absolute rectal bleeding subscore ≤1 at the end of induction. ^e^*P* value <.01. ^f^*P* value <.05.

**Table S10.** Odds ratio between filgotinib 200 mg and tofacitinib 10 mg on efficacy outcomes in the (A) biologic-naive and (B) biologic‑experienced subgroups during the induction period.

| **Filgotinib 200 mg vs tofacitinib 10 mg** | **Before matching** | | | **After matching** | | |
| --- | --- | --- | --- | --- | --- | --- |
|  | **OR** | **95% CI** | ***P* value^a^** | **OR** | **95% CI** | ***P* value^a^** |
| **Clinical remission^b^** | 1.0 | (0.5 to 2.1) | .99 | 1.0 | (0.5 to 2.2) | .93 |
| **Endoscopic improvement^c^** | 1.0 | (0.6 to 1.7) | .92 | 1.1 | (0.6 to 1.9) | .82 |
| **Clinical response^d^** | 0.8 | (0.5 to 1.2) | .32 | 0.8 | (0.5 to 1.3) | .37 |

A

B

| **Filgotinib 200 mg vs tofacitinib 10 mg** | **Before matching** | | | **After matching** | | |
| --- | --- | --- | --- | --- | --- | --- |
|  | **OR** | **95% CI** | ***P* value^a^** | **OR** | **95% CI** | ***P* value^a^** |
| **Clinical remission^b^** | 0.2 | (0.0 to 1.4) | .10 | 0.2 | (0.0 to 1.8) | .15 |
| **Endoscopic improvement^c^** | 0.5 | (0.2 to 1.4) | .20 | 0.7 | (0.3 to 2.0) | .54 |
| **Clinical response^d^** | 1.6 | (0.9 to 2.7) | .11 | 2.0 | (1.1 to 3.7) | **<.05^e^** |

Abbreviations: CI, confidence interval; OR, odds ratio.

^a^*P* values were calculated using the Wald test. ^b^Clinical remission is defined as the proportion of patients with total Mayo score ≤2, no subscore >1, and rectal bleeding of 0 at the end of induction. ^c^Endoscopic improvement is defined as the proportion of patients with endoscopic subscore ≤1 at the end of induction. ^d^Clinical response is defined as the proportion of patients with a reduction from baseline in total Mayo score ≥3 and ≥30%, and reduction from baseline in the rectal bleeding subscore ≥1 or absolute rectal bleeding subscore ≤1 at the end of induction. ^e^*P*value <.05.

**Table S11.** Odds ratio between filgotinib 200 mg and ustekinumab 6 mg/kg on efficacy outcomes in the (A) biologic-naive and (B) biologic-experienced subgroups during the induction period.

A

| **Filgotinib 200 mg vs ustekinumab 6 mg/kg** | **Before matching** | | | **After matching** | | |
| --- | --- | --- | --- | --- | --- | --- |
|  | **OR** | **95% CI** | ***P* value^a^** | **OR** | **95% CI** | ***P* value^a^** |
| **Clinical remission^b^** | 1.1 | (0.5 to 2.5) | .77 | 1.1 | (0.5 to 2.5) | .75 |
| **Endoscopic improvement^c^** | 0.6 | (0.4 to 1.0) | .05 | 0.6 | (0.4 to 1.0) | .06 |
| **Clinical response^d^** | 1.1 | (0.6 to 1.9) | .81 | 1.1 | (0.6 to 2.0) | .73 |

B

| **Filgotinib 200 mg vs ustekinumab 6 mg/kg** | **Before matching** | | | **After matching** | | |
| --- | --- | --- | --- | --- | --- | --- |
|  | **OR** | **95% CI** | ***P* value^a^** | **OR** | **95% CI** | ***P* value^a^** |
| **Clinical remission^b^** | 0.3 | (0.0 to 1.7) | .17 | 0.3 | (0.0 to 1.6) | .15 |
| **Endoscopic improvement^c^** | 0.9 | (0.3 to 2.3) | .75 | 0.8 | (0.3 to 2.1) | .59 |
| **Clinical response^d^** | 1.8 | (1.0 to 3.2) | **<.05^e^** | 1.8 | (1.0 to 3.3) | .06 |

Abbreviations: CI, confidence interval; OR, odds ratio.

^a^*P* values were calculated using the Wald test. ^b^Clinical remission is defined as the proportion of patients with total Mayo score ≤2 and no subscore >1 at the end of induction. ^c^Endoscopic improvement is defined as the proportion of patients with endoscopic subscore ≤1 at the end of induction. ^d^Clinical response is defined as the proportion of patients with a reduction from baseline in total Mayo score ≥3 and ≥30%, and reduction from baseline in the rectal bleeding subscore ≥1 or absolute rectal bleeding subscore ≤1 at the end of induction. ^e^*P* value <.05.

## Appendix 2: Comparative Corticosteroid-Free Clinical Remission Between Filgotinib and Tofacitinib

**Table S12.** Odds ratio between filgotinib 200 mg and tofacitinib 5 mg for corticosteroid-free clinical remission in the overall maintenance population.

| **Filgotinib 200 mg vs tofacitinib 5 mg** | **Before matching** | | | **After matching** | | |
| --- | --- | --- | --- | --- | --- | --- |
|  | **OR** | **95% CI** | ***P* value^a^** | **OR** | **95% CI** | ***P* value^a^** |
| **Corticosteroid-free clinical remission^b^** | 3.1 | (0.7 to 14.7) | .15 | 2.0 | (0.4 to 9.1) | .39 |

Abbreviations: CI, confidence interval; OR, odds ratio; Mayo clinical score.

^a^*P* values were calculated using the Wald test. ^b^Corticosteroid-free clinical remission using the SELECTION individual patient data was calculated as among the patients who were receiving corticosteroids at re-baseline, the proportion of those who discontinued corticosteroids and were in remission (MCS remission and rectal bleeding subscore of 0) at the end of the maintenance period. In OCTAVE SUSTAIN, corticosteroid-free clinical remission was defined as among the patients who were receiving corticosteroids at re-baseline, the proportion of those who were in remission (MCS remission and rectal bleeding subscore of 0) in addition to not requiring any treatment with corticosteroids for at least 4 weeks before the assessment.

## Appendix 3: Comparative Safety Between Filgotinib and Each Comparator in the Maintenance Phase

**Table** **S13.** Safety outcomes among the overall maintenance populations of filgotinib 200 mg vs vedolizumab IV 300 mg.

| **Filgotinib 200 mg vs vedolizumab IV** | **Before matching** | | | **After matching** | | |
| --- | --- | --- | --- | --- | --- | --- |
|  | **OR** | **95% CI** | ***P* value^a^** | **OR** | **95% CI** | ***P* value^a^** |
| Serious AE | 4.7 | (1.1 to 19.4) | **<.05^b^** | 11.8 | (2.5 to 54.8) | **<.01^c^** |
| Any infection | 1.5 | (0.8 to 2.6) | .18 | 1.6 | (0.8 to 3.0) | .19 |
|  | **RD** | **95% CI** | ***P* value^a^** | **RD** | **95% CI** | ***P* value^a^** |
| Serious infection | 1.7 | (–2.6 to 6.0) | .44 | 1.6 | (−2.7 to 5.9) | .46 |

Abbreviations: AE, adverse event; CI, confidence interval; IV, intravenous; OR, odds ratio; RD, risk difference.

^a^*P* values were calculated using the Wald test. ^b^*P* value <.05. ^c^*P* value <.01.

**Table S14.** Safety outcomes among the overall maintenance populations of filgotinib 200 mg vs vedolizumab SC 108 mg.

| **Filgotinib 200 mg vs vedolizumab SC** | **Before matching** | | | **After matching** | | |
| --- | --- | --- | --- | --- | --- | --- |
|  | **OR** | **95% CI** | ***P* value^a^** | **OR** | **95% CI** | ***P* value^a^** |
| Serious AE | 2.5 | (0.5 to 12.1) | .24 | 1.4 | (0.3 to 7.2) | .65 |
| AE leading to treatment discontinuation | 3.4 | (0.5 to 24.6) | .22 | 4.5 | (0.6 to 35.7) | .15 |

Abbreviations: AE, adverse event; CI, confidence interval; OR, odds ratio; SC, subcutaneous.

^a^*P* values were calculated using the Wald test.

**Table S15.** Safety outcomes among the overall maintenance populations of filgotinib 200 mg vs tofacitinib 5 mg.

| **Filgotinib 200 mg vs tofacitinib 5 mg** | **Before matching** | | | **After matching** | | |
| --- | --- | --- | --- | --- | --- | --- |
|  | **OR** | **95% CI** | ***P* value^a^** | **OR** | **95% CI** | ***P* value^a^** |
| AE leading to treatment discontinuation | 4.0 | (0.8 to 20.7) | .10 | 5.6 | (0.9 to 33.3) | .06 |
|  | **RD** | **95% CI** | ***P* value^a^** | **RD** | **95% CI** | ***P* value^a^** |
| Serious AE | 6.0 | (0.6 to 11.4) | **<.05^b^** | 6.2 | (0.6 to 11.8) | **<.05^b^** |
| Serious infection | 1.0 | (−1.4 to 3.4) | .42 | 0.8 | (−1.5 to 3.0) | .51 |
| Herpes zoster | −0.5 | (−2.7 to 1.7) | .65 | −0.8 | (−2.8 to 1.2) | .42 |

Abbreviations: AE, adverse event; CI, confidence interval; OR, odds ratio; RD, risk difference.

^a^*P* values were calculated using the Wald test. Because there were no patients with serious AEs, serious infections, or herpes zoster in the placebo arm of the SELECTION trial, it was not possible to estimate the OR. The RD was therefore calculated for these outcomes. ^b^*P*value <.05.

**Table S16.** Safety outcomes among the overall maintenance populations of filgotinib 200 mg vs ustekinumab 90 mg.

| **Filgotinib 200 mg vs ustekinumab  90 mg Q8W** | **Before matching** | | | **After matching** | | |
| --- | --- | --- | --- | --- | --- | --- |
|  | **OR** | **95% CI** | ***P* value^a^** | **OR** | **95% CI** | ***P* value^a^** |
| AE leading to treatment discontinuation | 7.7 | (1.2 to 47.7) | **<.05^b^** | 6.2 | (0.8 to 50.6) | .09 |
| Any infection | 1.4 | (0.9 to 2.4) | .16 | 1.5 | (0.9 to 2.6) | .15 |
|  | **RD** | **95% CI** | ***P* value^a^** | **RD** | **95% CI** | ***P* value^a^** |
| Serious AE | 5.6 | (−1.0 to 12.3) | .10 | 5.3 | (−1.4 to 11.9) | .12 |
| Serious infection | 1.6 | (−1.7 to 4.8) | .34 | 1.3 | (−1.8 to 4.3) | .42 |

Abbreviations: AE, adverse event; CI, confidence interval; OR, odds ratio; Q8W, every 8 weeks; RD, risk difference.

^a^*P* values were calculated using the Wald test. Because there were no patients with serious AEs or serious infections in the placebo arm of the SELECTION trial, it was not possible to estimate the OR. The RD was therefore calculated for these outcomes. ^b^*P* value <.05.

## Appendix 4: Comparative Health-Related Quality of Life Between Filgotinib and Each Comparator in the Maintenance Phase

### Filgotinib vs Vedolizumab

Improvement in Inflammatory Bowel Disease Questionnaire (IBDQ) and 36-item Short-Form Heath Survey (SF-36) outcomes were similar between filgotinib 200 mg and intravenous vedolizumab 300 mg (**Table S17**). Significant differences (*P* < .05) or differences close to significance (*P* = .05) were observed between the placebo groups in SELECTION and GEMINI 1 for all outcomes.

When compared with subcutaneous vedolizumab 108 mg, filgotinib showed a significantly lower IBDQ total score improvement from induction baseline (difference, −31.4; 95% confidence interval [CI], −46.8 to −16.0; *P* < .001; **Table S18**). However, in the VISIBLE 1 publication, arm-level data were not reported; therefore, it was not possible to verify if the placebo arms were comparable after matching.

### Filgotinib vs Tofacitinib

Improvements in IBDQ outcomes were similar with filgotinib 200 mg and tofacitinib 5 mg (**Table S19**). Significant differences (*P* < .001) were observed between the placebo groups in SELECTION and OCTAVE SUSTAIN for both outcomes.

### Filgotinib vs Ustekinumab

Improvements in IBDQ and SF-36 outcomes were mostly similar with filgotinib 200 mg and ustekinumab 90 mg, with the exception of SF-36 Physical Component Summary (odds ratio, 0.6; 95% CI, 0.3 to 1.0; *P* < .05; **Table S20**). Significant differences (*P* < .001) were observed between the placebo groups in SELECTION and UNIFI for all outcomes.

**Table** **S17.** HRQoL outcomes among the overall maintenance populations of filgotinib 200 mg vs vedolizumab IV 300 mg.

| **Filgotinib 200 mg vs vedolizumab IV** | **Before matching** | | | **After matching** | | |
| --- | --- | --- | --- | --- | --- | --- |
|  | **Difference** | **95% CI** | ***P* value^a^** | **Difference** | **95% CI** | ***P* value^a^** |
| IBDQ total score change between induction baseline^b^ and end of maintenance | −11.4 | (−20.8 to −1.9) | **<.05^c^** | −10.8 | (−24.3 to 2.8) | .12 |
|  | **OR** | **95% CI** | ***P* value^a^** | **OR** | **95% CI** | ***P* value^a^** |
| IBDQ total score ≥170 points at the end of maintenance | 0.8 | (0.5 to 1.4) | .53 | 1.4 | (0.8 to 2.6) | .28 |
| IBDQ total score change between induction baseline and end of maintenance ≥16 points | 0.8 | (0.3 to 1.8) | .59 | 1.3 | (0.5 to 3.3) | .64 |
| SF-36 PCS change between induction baseline and end of maintenance ≥5 points | 0.7 | (0.4 to 1.1) | .14 | 0.7 | (0.4 to 1.4) | .34 |
| SF-36 MCS change between induction baseline and end of maintenance ≥5 points | 0.9 | (0.6 to 1.6) | .83 | 0.9 | (0.5 to 1.7) | .84 |

Abbreviations: CI, confidence interval; HRQoL, health-related quality of life; IBDQ, Inflammatory Bowel Disease Questionnaire; IV, intravenous; MCS, Mental Component Summary; OR, odds ratio; PCS, Physical Component Summary; SF-36, 36-item Short-Form Heath Survey; TNF, tumor necrosis factor.

^a^*P* values were calculated using the Wald test. ^b^Adjusted mean change from baseline was estimated using analysis of covariance with adjustment for 2 stratification factors to be consistent with GEMINI 1: a) concomitant use or non-use of corticosteroids, and b) concomitant use or non-use of immunosuppressive agents or previous use or non-use of anti-TNF agents. Missing data for IBDQ scores at end of maintenance were imputed using the last observation carried forward approach. ^c^*P* value <.05.

**Table** **S18.** HRQoL outcomes among the overall maintenance populations of filgotinib 200 mg vs vedolizumab SC 108 mg.

| **Filgotinib 200 mg vs vedolizumab SC** | **Before matching** | | | **After matching** | | |
| --- | --- | --- | --- | --- | --- | --- |
|  | **Difference** | **95% CI** | ***P* value^a^** | **Difference** | **95% CI** | ***P* value^a^** |
| IBDQ total score change from induction baseline^b^ | −33.1 | (−48.3 to −17.9) | **<.001^c^** | −31.4 | (−46.8 to −16.0) | **<0.001^c^** |

Abbreviations: CI, confidence interval; HRQoL, health-related quality of life; IBDQ, Inflammatory Bowel Disease Questionnaire; SC, subcutaneous.

^a^*P* values were calculated using the Wald test.  ^b^IBDQ total score change between induction baseline and end of maintenance was adjusted using an analysis of covariance model with baseline scores as a covariate. Missing data for IBDQ scores at the end of maintenance were imputed using the last observation carried forward approach. These approaches were in line with VISIBLE 1. ^c^*P* value <.001.

**Table** **S19.** HRQoL outcomes among the overall maintenance populations of filgotinib 200 mg vs tofacitinib 5 mg.

| **Filgotinib 200 mg vs tofacitinib 5 mg** | **Before matching** | | | **After matching** | | |
| --- | --- | --- | --- | --- | --- | --- |
|  | **OR** | **95% CI** | ***P* value^a^** | **OR** | **95% CI** | ***P* value^a^** |
| IBDQ total score ≥170 points at the end of maintenance | 0.5 | (0.3 to 0.9) | **<.05^b^** | 0.7 | (0.4 to 1.1) | .12 |
| IBDQ total score change between induction baseline and end of maintenance ≥16 points | 0.6 | (0.3 to 1.2) | .16 | 0.6 | (0.3 to 1.4) | .25 |

Abbreviations: CI, confidence interval; HRQoL, health-related quality of life; IBDQ, Inflammatory Bowel Disease Questionnaire; OR, odds ratio.

^a^*P* values were calculated using the Wald test. ^b^*P* value <.05.

**Table S20.** HRQoL outcomes among the overall maintenance populations of filgotinib 200 mg vs ustekinumab 90 mg.

| **Filgotinib 200 mg vs ustekinumab  90 mg Q8W** | **Before matching** | | | **After matching** | | |
| --- | --- | --- | --- | --- | --- | --- |
|  | **OR** | **95% CI** | ***P* value^a^** | **OR** | **95% CI** | ***P* value^a^** |
| IBDQ total score change between induction baseline and end of maintenance ≥16 points | 0.7 | (0.3 to 1.6) | .40 | 0.8 | (0.4 to 1.9) | .67 |
| SF-36 MCS change between induction baseline and end of maintenance ≥5 points | 0.5 | (0.3 to 0.8) | **<.01^b^** | 0.6 | (0.4 to 1.0) | .07 |
| SF-36 PCS change between induction baseline and end of maintenance ≥5 points | 0.6 | (0.3 to 0.9) | **<.05^c^** | 0.6 | (0.3 to 1.0) | **<.05^c^** |

Abbreviations: CI, confidence interval; HRQoL, health-related quality of life; IBDQ, Inflammatory Bowel Disease Questionnaire; OR, odds ratio; Q8W, every 8 weeks; SF-36, 36-item Short-Form Health Survey; MCS, Mental Component Summary; PCS, Physical Component Summary.

^a^*P* values were calculated using the Wald test. ^b^*P* value <.01. ^c^*P* value <.05.
